# A synthetic expression system for orthogonal gene expression in *Nicotiana benthamiana*

**DOI:** 10.1007/s11103-026-01725-7

**Published:** 2026-06-02

**Authors:** Dominik Mojzita, Anssi Rantasalo, Markus Laurel, Hannu Hotti, Kirsi-Marja Oksman-Caldentey, Heiko Rischer

**Affiliations:** 1 VTT Technical Research Centre of Finland Ltd, P.O. Box 1000, 02044 Espoo, Finland; 2 EniferBio Oy, Espoo, Finland; 3 University of Turku, Turku, Finland

**Keywords:** Core promoter, Plant biotechnology, Recombinant protein production, Synthetic biology, Synthetic expression system, Transient expression

## Abstract

Plants represent commercially relevant production systems for recombinant proteins and chemical compounds. Effective genetic engineering depends on precise control of heterologous gene expression, which remains challenging due to complex transcriptional and post-transcriptional regulation, endogenous gene silencing mechanisms, and notably because of limited number of tools for allowing robust, fine-tuned control of expression levels across different systems/organisms. Some of the most common issues associated with plant expression systems are addressed with our plant-optimized version of a previously developed fungal universal synthetic expression system (SES). Plant SES demonstrates several favorable characteristics for robust heterologous gene expression, including highly constitutive function with apparently reduced sensitivity to endogenous silencing in transient assays, without requiring p19 co‑expression under the tested conditions. These together provide simple, predictable tuning of gene expression levels, and the potential for very high expression levels of the target genes. In all these features, SES shows higher and more stable transcript levels than Cauliflower Mosaic Virus (CaMV) 35 S promoter-based constructs in our experimental setups. The functionality of plant SES was tested by expressing mCherry and three commercially relevant proteins: fungal glucose oxidase (GOX), protein A and human vascular endothelial growth factor 165 (VEGF16) from diverse organisms, supporting high-level accumulation of recombinant proteins. In addition, plant SES retains full functionality in both plant and fungal hosts, which makes this expression system a useful tool for a multitude of genetic engineering applications in other eukaryotic organisms.

**Supplementary Information:**

The online version contains supplementary material available at 10.1007/s11103-026-01725-7.

## Key message

Plant synthetic expression system (SES) enables tunable transient expression of genes with high recombinant protein yields in *Nicotiana benthamiana* with the capability of inter-kingdom functionality.

**Supplementary Information:**

The online version contains supplementary material available at 10.1007/s11103-026-01725-7.

## Introduction

Synthetic biology has revolutionized the pharmaceutical, agricultural and biotechnology industries by applying engineering principles to biological systems and their component parts. The utilization of plants for this purpose was originally described as molecular pharming because the first examples focused on the production of pharmaceutically relevant molecules, such as antibodies (Hiatt et al. 1989) and blood proteins (Sijmons et al. 1990). Plants have been used to produce a wide variety of antibodies, enzymes, vaccines, and secondary metabolites, but most commercial products are non-pharmaceutical proteins due to the shorter approval process and lower production costs (Hood and Howard 2014). One of the key challenges in plant genetic engineering is the precise, robust and modular control of gene expression, and synthetic expression systems designed to regulate gene expression in a controlled manner, have contributed to addressing these issues (Huang et al. 2021; Malaquias et al. 2021).

Synthetic expression systems typically comprise a combination of synthetic promoters, transcription factors (TFs), and regulatory elements engineered to respond to specific inputs or environmental cues. These systems can be tailored to achieve tissue-specific expression, inducible responses, and graded gene expression levels (Huang et al. 2021). The integration of genetic circuitry into synthetic expression systems allows complex regulatory networks to be programmed, including dynamic gene expression in response to external signals (Andres et al. 2019; Belcher et al. 2020; Brophy et al. 2022). Orthogonal gene expression is the ability to regulate multiple genes independently without interference from endogenous plant regulatory elements.

The precise regulation of gene expression requires a promoter region that recruits TFs and RNA polymerase to initiate transcription (Porto et al. 2014; Villao-Uzho et al. 2023). Synthetic promoters are composed of a core promoter (CP), containing generic elements such as a TATA box and transcriptional start site, plus upstream regulatory elements that bind specific TFs (Liu and Stewart 2016). Synthetic promoters have enabled the development of orthogonal expression systems by avoiding interference with native gene regulation, thus reducing the overall metabolic burden (Brückner et al. 2015; Jores et al. 2021; Rushton et al. 2002). Synthetic transcription factors (sTFs) can be designed to target specific promoters or enhancers, enabling the construction of complex gene circuits that can respond to multiple inputs or modulate the activity of multiple genes simultaneously. For example, sTFs can be used to build feedback loops (Kar et al. 2022; McCarthy and Medford 2020) or logic gates that perform Boolean operations based on the presence or absence of specific input signals (Anderson et al. 2023; Andres et al. 2019; Ferreira and Antunes 2024).

Most synthetic expression systems are designed to function in narrow taxonomic groups, hindering the establishment of new production hosts. Universal systems that are decoupled from endogenous regulation should function across a broader spectrum of hosts. Recently, we established a universal synthetic expression system (SES) suitable for a wide range of fungi (Rantasalo et al. 2018a). The SES system is based on two expression cassettes, one driving the low-level constitutive expression of a synthetic transcription factor (sTF) by using a universal CP with basal transcriptional activity, and the second facilitates strong and tuneable expression of the target gene through a synthetic promoter composed of sTF-binding sites positioned upstream of another universal CP. Expression can be tuned by varying the number of sTF-binding sites.

Here we describe an optimized SES incorporating *Arabidopsis thaliana* CPs to make it compatible with the well-established plant host *Nicotiana benthamiana*. The robust functionality of the plant SES was validated by confirming the strong, constitutive, and tunable expression of a reporter gene and the expression of three industrially relevant proteins representing diverse protein classes. Despite recent developments in synthetic promoters in plants, relatively few expression systems have been shown to operate with high efficiency and tunability across distinct eukaryotic kingdoms.

## Materials and methods

### Strains and media

*Escherichia coli* (TOP10) was used for DNA cloning, *Saccharomyces cerevisiae* strain H4489 (expressing the sTF LexA-VP16) for the *A. thalian*a CP screen, *S. cerevisia*e strain CEN.PK111-32D (provided by Dr. P. Kötter, Institute of Microbiology, J.W. Goethe University, Germany) for testing selected plant SES variants in yeast, and *Rhizobium radiobacter* (*Agrobacterium tumefacien*s) strain EHA105 (Hood et al. 1993) for leaf infiltration in *N. benthamiana*.

Bacteria were cultivated in lysogeny broth (LB) comprising 10 g L^−1^ tryptone, 5 g L^−1^ yeast extract (Becton Dickinson) and 0.17 M NaCl, supplemented with 100 µg mL^−1^ ampicillin (*E. coli*) or 10 µg mL^−1^ rifampicin and 50 µg mL^−1^ kanamycin (*R. radiobacter*). Yeast was cultivated in SCD medium without uracil, comprising 6.7 g L^−1^ yeast nitrogen base (Becton Dickinson), 20 g L^−1^ d-glucose and 790 mg L^−1^ complete supplement mixture without uracil (Formedium).

*N. benthamiana* seeds were sown in round 5-cm pots made from compressed peat using standard peat-based germination soil containing perlite. Seeds were germinated in our greenhouse facility at 24 ± 1 °C and 60% humidity with a 16-h photoperiod (provided by 36-W cool white, fluorescent lamps) and manual watering without added fertilizers. After 1 week, seedlings were transferred to a nutrient film technique watering system with watering cycles of 15 min four times daily providing half-strength hydroponic fertilizers (HaifaCAL NPK 15,5-0-0-Ca with Hyperflex-1 NPK 8-6-30) under 400-W high-pressure sodium lamps. During weeks 2–3, seedlings were thinned down to one per pot, and on the fourth week they were provided with full-strength fertilizers.

### DNA cloning

All vectors were cloned by Gibson assembly (New England Biolabs, Ipswich, USA) or restriction/ligation (Thermo Fisher Scientific, Bellefonte, USA). Kapa Hi-Fi DNA polymerase (Kapa Biosystems) was used for all PCR steps. Plasmids assembled in vitro were introduced into *E. coli* TOP10 cells by electroporation and verified by analytical digestion and sequencing. Synthetic DNA fragments and primers were obtained from Integrated DNA Technologies (IDT, Newark, USA), which also codon optimized the sequences for *N. benthamiana* using the online Codon Optimization Tool (sequence type = amino acids; product type = gBlocks; organism = *N. benthamiana*). The cloning strategy is outlined in Fig. S1a–e. The DNA sequences of the CPs, primers, genes and other sequences are listed in Tables S1–S3.

### CP screen and selection of the CP candidates

**Fig. 1:**
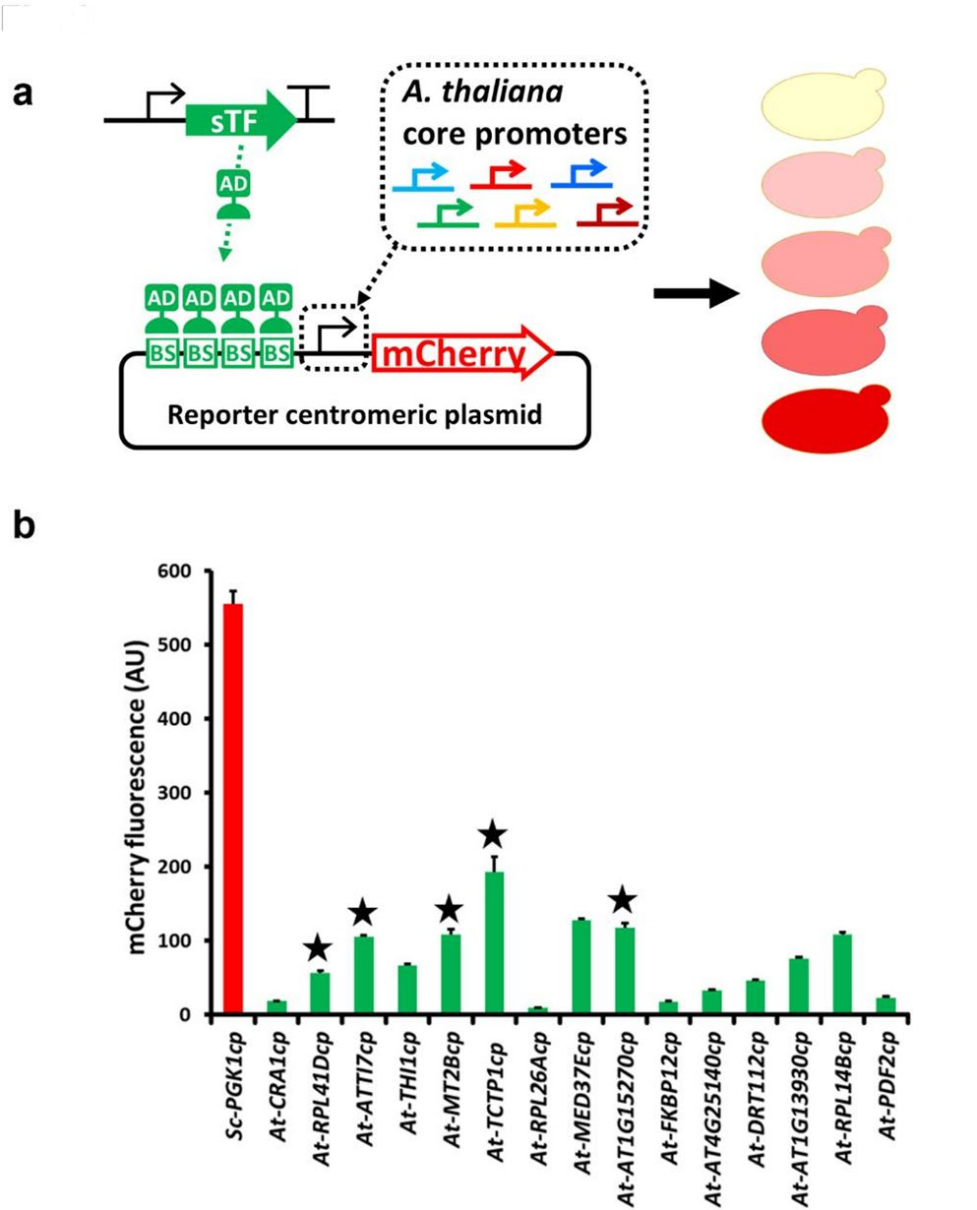
**a** Core promoter screen in *Saccharomyces cerevisiae*. Schematic representation of the core promoter (CP) screen in *S. cerevisiae*. A yeast strain constitutively expressing a synthetic transcription factor (sTF) was transformed with reporter centromere plasmids containing diverse CPs originating from strongly expressed genes in *A. thaliana*. The reporter plasmids were assembled in vivo with the candidate CPs to form sTF-dependent mCherry expression cassettes. The resulting yeast strains were tested for red fluorescence to confirm each CP was functional (able to trigger transcription when activated by the upstream-bound sTF) and for the level of activity. **b** *A. thaliana* core promoter screen in *S. cerevisiae*. Five CPs (asterisks) were selected for the construction of an *N. benthamiana* synthetic expression system (SES). The native yeast *PGK1* core promoter (red bar) was used as a positive control. Data are means + standard deviations from *n* = 3 independent cultures

The functionality of CPs from selected *A. thaliana* genes was screened in *S. cerevisiae* (Rantasalo et al. 2018a). Briefly, ~ 300 candidate CPs from strongly expressed genes (Wickramasuriya and Dunwell 2015) were manually inspected, and those with a potential TATA box 120–150 bp upstream of the start codon and a well-defined transcriptional start site paired with a short 5′ untranslated region (5′-UTR), were selected. The 15 most promising candidates included a region up to 50 bp upstream of the presumed TATA box and the *Pac*I restriction site (with an extra adenine base) introduced immediately upstream of the start codon (Table S1). The CPs were obtained as synthetic DNA with 55-bp homology regions to allow sTF-dependent reporter plasmid assembly in vivo by homologous recombination in *S. cerevisiae* constitutively expressing sTF LexA-VP16 (Rantasalo et al. 2018a) (Fig. 1a).

Pooled *S. cerevisiae* transformants were cultivated and the cell suspensions were tested for mCherry fluorescence with measurement intervals of 500 ms to determine the strength of each CP in yeast (Fig. 1b). Varioskan instrument (Thermo Fisher Scientific, Bellefonte, USA) was used as previously described, with fluorescence values normalized against the OD_600_ (Rantasalo et al. 2018a). The excitation and emission wavelengths were 587 and 610 nm, respectively, and the excitation bandwidth was 5 nm.

**Fig. 2:**
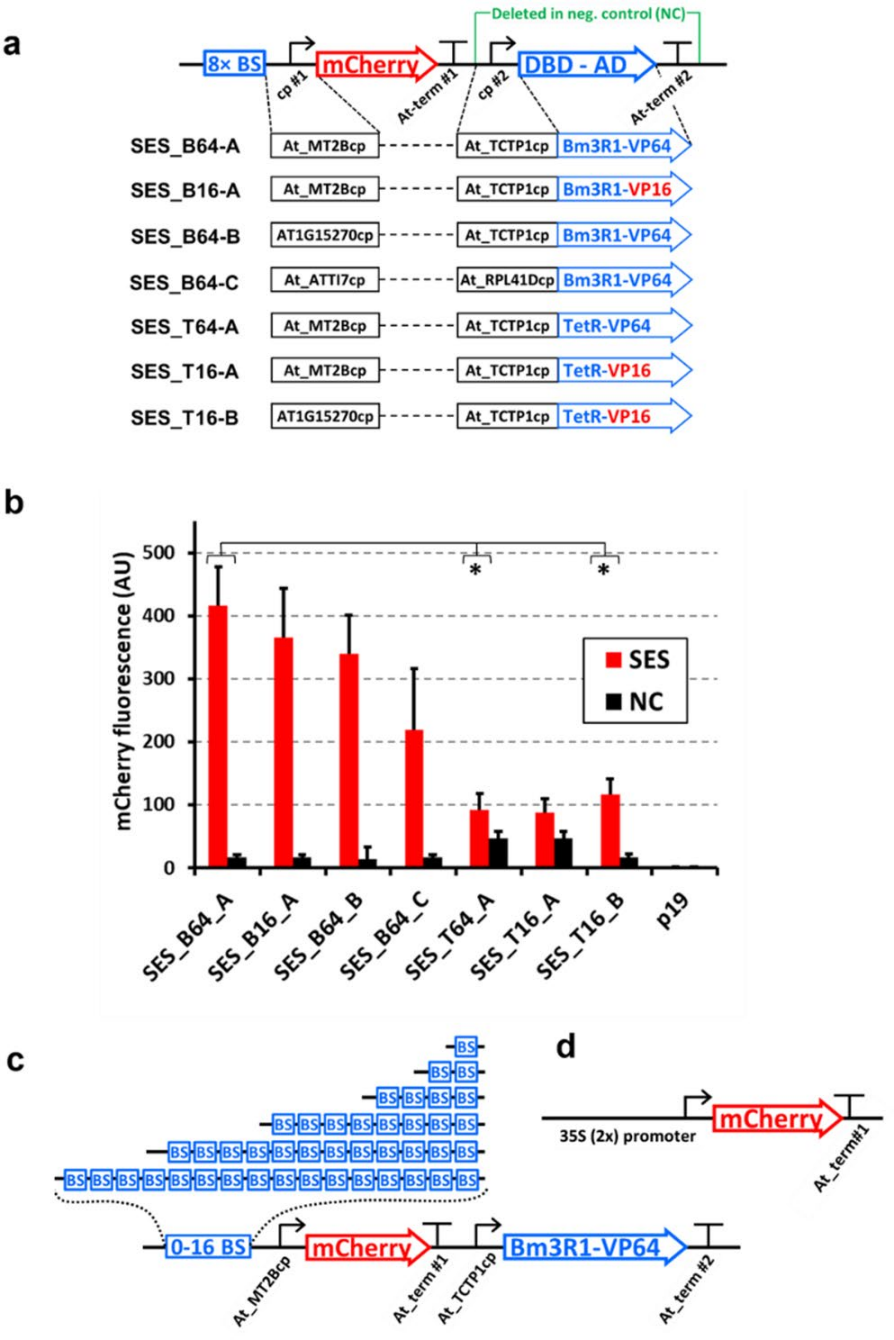
**a** Evaluation of DNA parts for the synthetic expression system. Schematic representation of the seven SES versions we tested. The expression of mCherry was controlled by synthetic promoters containing selected CPs and eight upstream sTF-binding sites. The mCherry reporter cassettes were linked to sTF cassettes driven by another CP based on either the Bm3R1 or TetR DNA-binding domain (DBD). The sTFs contained either a VP16 or VP64 transcriptional activation domain. The negative controls (NC) for each version lacked the sTF expression cassette. The terminators originated from *A. thaliana* (Table S2). Each SES was transferred to an *R. radiobacter* vector for transient expression in tobacco leaves. **b** Fluorescence in the leaf extracts. Fluorescence intensity was normalized to the protein concentration in the leaf extracts. SES_B64-A was selected for further use, featuring the Bm3R1-based sTF with the VP64 activation domain and *A. thaliana* CPs *MT2B* and *TCTP1*. Data are means + standard deviations from *n* = 3 independent extracts prepared 6 days post-infiltration, and p19 represents a control infiltrated only with the *R. radiobacter* strain carrying the p19 cassette. A Shapiro-Wilk test indicated normal distribution for samples (*n* = 4). Respective pairwise comparison of SES_B64_A with SES_T64_A or SES_T16_B using a two-tailed Student’s t-test showed a significant difference (*P < 0.05). **c** Schematic representation of the SES constructs based on version SES_B64-A. Seven alternatives were constructed containing different numbers of sTF (Bm3R1) binding sites (0–16). **d** Schematic representation of the control construct for mCherry expression containing the CaMV 2 × 35 S promoter. The mCherry coding region, terminator and vector backbone were identical to the constructs in panel (c)

Five CPs were selected for the plant SES (Table S1). The first two (At-RPL41Dcp and At-ATTI7cp) were chosen based on subjective assessment, resulting in the “first generation” plant SES later named SES-B64C (Fig. 2a). The other three were chosen from 13 candidates based on fluorescence levels in yeast, resulting in a “second generation” plant SES where different DNA-binding and activation domains were tested (Fig. 2b). The most active CP in the yeast screen (At-TCTP1cp) was used in the sTF expression cassette of the plant SES due to the higher likelihood of high basal transcription, which is necessary for sufficient sTF expression.

### Infiltration of *N. benthamiana* leaves

The *R. radiobacter* strains carrying SES expression vectors were grown in LB overnight and the OD_600_ was adjusted to 0.8 with infiltration buffer (1 mM MES, 1 mM MgSO_4_, pH 5.5). The suspension was used alone or mixed 1:1 with another strain carrying the p19 helper vector (Lakatos et al. 2004; Silhavy et al. 2002) at the same OD_600_. The p19 helper suspension was also used alone as an infiltration control. Young and fully formed leaves of 5–6-week‐old *N. benthamiana* plants were infiltrated from the abaxial surface using a syringe. The infiltrated plants were kept in darkness overnight, then cultivated in the greenhouse for up to 7 days as described above.

### Whole-plant images

Plants were transferred to a dark room for imaging using a Canon EOS 650D digital camera without filters (ambient light settings) or a red filter (Cokin P.003) mounted on the lens for the visualization of red fluorescence. In the latter case, plants were illuminated (Schott KL2500LCD) using a green excitation filter (λ = 540 nm).

### Leaf tissue sample preparation

For each sample (collected at least in triplicate), four discs (Ø 7.1 mm) were cut from the infiltrated areas of the leaves and collected into 2-mL tubes containing 4 × 2.3-mm zircon beads (BioSpec, Bartlesville, USA). The tubes were transferred to liquid nitrogen and stored at − 80 °C. Before analysis, the frozen samples were powdered in a Retsch ball mill (2 × 1-min pulse at 29 Hz) (Retsch, Haan, Germany).

**Fig. 3:**
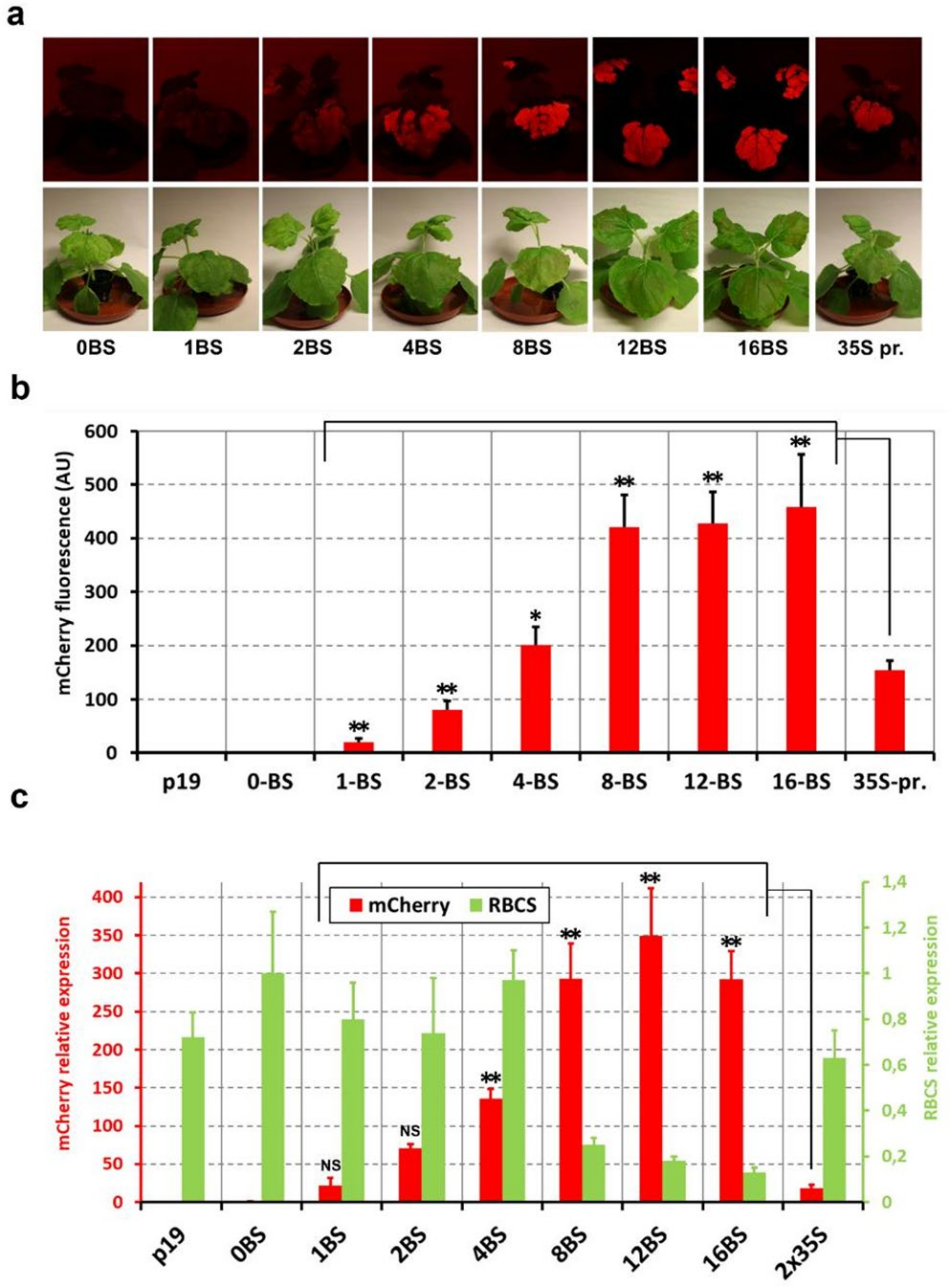
**a** Development of a tunable SES for a broad range of expression levels. Representative images of *N. benthamiana* plants infiltrated with a specific version of the SES (Fig. 2c) or a control construct of 2 × 35 S (Fig. 2d) along with the p19 expression cassette. The photographs were taken 6 days post-infiltration under conditions for the visualization of red fluorescence (top panel) or in bright light (bottom panel). **b** Fluorescence in the leaf extracts. Fluorescence intensity was normalized to the protein concentration in the leaf extracts. Data are means + standard deviations (*n* = 10). A Shapiro-Wilk test indicated normal distribution for all samples. Pairwise comparisons of fluorescence values of each SES construct (1–16 binding sites) with the 2 × 35 S promoter using one way ANOVA with Tukey post-hoc test and Holm-Bonferroni comparison. Statistical significance is indicated by asterisks *P < 0.05, **P < 0.01. **c** Transcription analysis. The relative gene expression levels of mCherry and RBCS was calculated using the using the 2^−ΔΔCT^ method with 0BS construct as a control and using the constitutive *UBC-2e* gene as a reference. Data are means + standard deviations of two biological replicates (two different plants) and at least four technical replicates. A Shapiro-Wilk test indicated normal distribution for all samples. Pairwise comparisons of relative expression of each SES construct (1–16 BS) with the 2 × 35 S promoter using one way ANOVA with Tukey post-hoc test and Holm-Bonferroni comparison. Statistical significance is indicated by asterisks **P < 0.01, NS = no significant difference

For mCherry fluorescence (Fig. 3b), 0.6 mL PBS (150 mM NaCl, 12 mM Na_2_HPO_4_, 3 mM NaH_2_PO_4_, pH 7.3) containing Roche Complete protease inhibitors (Roche, Basel, Switzerland), was added. The sample was then mixed by vortexing, the debris was pelleted, (15,000 g, 20 min, 4 °C) and transferred 200 µL of supernatant to Black 96-well Cliniplates (Thermo Fisher Scientific, Bellefonte, USA) for measurement using a Varioskan instrument as described above for mCherry detection. The total protein concentration was determined using a Bradford assay (Bio-Rad, Hercules, USA).

Total RNA was purified using the RNeasy Plant Mini Kit (Qiagen, Venlo, Netherlands). Samples were mixed with 0.6 mL of RLT buffer plus β-mercaptoethanol before transferring 450 µL of the extract to a QIAshredder spin column. Total RNA was eluted in 50 µL RNase-free water, and its concentration and purity were determined using a Nanodrop 2000 spectrophotometer (Thermo Fisher Scientific, Bellefonte, USA) before storage at − 80 °C.

For protein extraction (Fig. 5), samples were mixed with 0.6 mL 1x SDS loading buffer (100 mL L^−1^ glycerol, 25 mL L^−1^ β-mercaptoethanol, 0.5 g L^−1^ OrangeG dye (Sigma-Aldrich, St. Louis, USA), 10 g L^−1^ SDS, and 31.2 mM Tris-HCl, pH 6.8) before boiling. The debris was then pelleted by centrifugation (15,000 g, 20 min, 4 °C) and the supernatant was loaded onto a 4–20% SDS-PAGE gradient gel (BioRad, Hercules, USA).

For TBS extraction and protein purification (Fig. 5), several *N. benthamiana* leaves 6 days post-infiltration were ground to powder in a porcelain mortar containing liquid nitrogen before adding 50 mL TBS (100 mM Tris, 150 mM NaCl, 1 mM EDTA, pH 8.0) containing Roche complete protease inhibitors. The mixture was centrifuged (15,000 g, 20 min, 4 °C) to pellet the debris and 50 µL of the supernatant was mixed with 20 µL 4x SDS-loading buffer before analysis by SDS-PAGE as above. The rest of the supernatant was used for STREP-II tag protein purification.

### cDNA synthesis and quantitative RT-PCR

First-strand cDNA was synthesized from 1 µg of total RNA which was pretreated with RNase-free DNase I, (Thermo Fisher Scientific, Bellefonte, USA) using anchored-oligo(dT)_18_ primers and the first-strand cDNA synthesis kit (Roche, Basel, Switzerland). The cDNA was amplified by PCR in white LightCycler 480 96-well plates on a Lightcycler 480II instrument (Roche, Basel, Switzerland) by combining 2.5 µL 100x diluted cDNA samples, 2.5 µl qPCR primer mix (8 µM each primer), and 15 µl 1.5x diluted LightCycler 480 SYBR Green I Master Mix (Roche). The relative gene expression of constructs was calculated using the 2^−ΔΔCT^ method (Livak and Schmittgen 2001) using the 0BS construct with basal core promoter activity as a control to calculate fold changes in expression. The expression levels of the ribulose bisphosphate carboxylase (RuBisCo) small chain gene (RBCS), mCherry and sTF (Bm3R1) were normalized to the low but stably expressed gene encoding ubiquitin-conjugating enzyme e2-10 (UBC-e2). The primers, target gene IDs in the *N. benthamiana* genome database and the length of each RT-PCR amplicon are shown in Table S4.

### Purification of recombinant proteins

Recombinant proteins were isolated from TBS extracts on gravity flow columns packed with 1 mL Strep-Tactin Superflow high-capacity resin (IBA Lifesciences, Göttingen, Germany). The resin was equilibrated with 2 mL TBS (buffer W: 100 mM Tris, 150 mM NaCl, 1 mM EDTA, pH 8.0). The TBS extracts (~ 20 mL) were loaded onto the columns, and bound proteins were washed with 4 × 2 mL TBS before elution in 4 × 0.5 mL elution buffer (TBS/buffer W with 2.5 mM desthiobiotin). Fractions 2–4 were collected, and the protein concentration was determined using a Bradford assay (Bio-Rad Protein Assay, Hercules, USA). 50 µL of each eluent was mixed with 20 µL 4x SDS-loading buffer before boiling, centrifugation and SDS-PAGE analysis as described above.

### Western blot analysis

Samples were loaded together with a dilution series of mCherry (with C-terminal STREP-II tag) and fractionated on 4–20% SDS-PAGE gradient gels (BioRad, Hercules, USA). The proteins were transferred onto a nitrocellulose membrane using the Trans-Blot Turbo system (BioRad, Hercules, USA), and the membranes were blocked with Odyssey blocking solution (LI-COR Biosciences, Lincoln, USA). Proteins with STREP-II tags were detected with the Strep-Tactin AP conjugate (IBA Lifesciences, Göttingen, Germany) diluted 1:2000 in TBST (10 mM Tris-HCl, 4.4 g L^−1^ NaCl, 0.5 mL L^−1^ Tween-20, pH 8.0). The signal was detected by incubating the membranes with BCIP/NBT Color Development Substrate (Promega, Madison, USA) in AP-buffer (10 mM Tris-HCl, 100 mM NaCl, 1 mM MgCl_2_, pH 9.5). The membranes were scanned using a GS-710 calibrated imaging densitometer (BioRad) and signals were quantified using Quantity One software (BioRad, Hercules, USA).

### Coomassie staining

Samples were loaded on 4–20% SDS-PAGE gradient gels together with dilutions of the following commercially available proteins: glucose oxidase (Sigma-Aldrich, St. Louis, USA), protein A (Pierce Biotechnology, Rockford, USA) or VEGF165 (ORF Genetics, Kopavogur, Iceland). The gels were immersed in a colloidal Coomassie stain (PageBlue Protein Staining Solution; Thermo Fisher Scientific, Bellefonte, USA) according to the manufacturer’s protocol. The stained gels were scanned using the Odyssey CLx Imaging System (LI-COR Biosciences, Lincoln, USA) and the signals were quantified using Image Studio (LI-COR Biosciences, Lincoln, USA).

### Protein quantification

Proteins were quantified by the densitometric analysis of western blots. However, to avoid inaccuracy caused by the limited dynamic range of the signal and any protein-specific effects on the detection of the STREP-II tag, secondary quantification was included, based on Coomassie staining. The results of the two methods were similar, but the results were reported as estimates, rather than exact values. The percentage of total soluble protein (TSP) was calculated from the signals obtained from TBS extracts, whereas absolute concentrations (g/kg) were estimated from the SDS extracts. The absolute concentrations (and both values for VEGF165) were calculated only from the western blots because there was a strong background on the Coomassie-stained gels. This approach is illustrated using fungal glucose oxidase (GOX) as an example in Fig. S5.

### Functional analysis of the recombinant proteins

Glucose oxidase (GOX) activity was analyzed using a K-GLOX glucose oxidase assay kit (Megazyme, Wicklow, Ireland) according to the manufacturer’s instructions (microplate assay procedure). Commercially available glucose oxidase (Sigma-Aldrich, St. Louis, USA) was used as a positive control. Dilution series were prepared with a GOX standard (Megazyme, Wicklow, Ireland), GOX isolated from *N. benthamiana* (SES-GOX), and commercial GOX (Sigma-Aldrich, St. Louis, USA) as a positive control. The reactions were kept at 25 °C for 20 min, and DAbs510 was recorded for all dilutions. The standard curve was calculated, and DAbs510 values of the test GOX samples, which fitted the standard range, were used to calculate U/mg values. Each sample was tested in duplicate.

Protein A binding to IgG was analyzed using a real time quartz crystal microbalance with dissipation monitoring (QCM-D) technique based on the QSense Analyzer E4 and QSense polystyrene sensors QSX 305 PS (Biolin Scientific, Gothenburg, Sweden) as previously reported (Kurppa et al. 2018). The sensors were equilibrated with 100 mM phosphate buffer (pH 7.0), and the same buffer was used for washing and as the solvent for all protein samples. The sensors were cleaned, mounted into the running chambers, and equilibrated according to the manufacturer’s instructions. The flow was set and maintained at 0.1 mL/min throughout the experiment. After equilibration, the protein samples were allowed to flow across the sensor for 10–20 min followed by a ~ 15-min wash. The samples were 0.1 mg mL^−1^ protein A isolated from *N. benthamiana* (SES-protA), commercial standard protein A (Pierce), and BSA as a negative control. IgG from human serum 0.01 mg mL^−1^ (Sigma-Aldrich, St. Louis, USA) was then passed across the sensor for ~ 15 min, followed by a ~ 20-min wash. The protein A–IgG interaction was distributed by introducing glycine-HCl buffer (pH 2.2) for ~ 10 min, followed by a ~ 20-min wash. The quantity of bound IgG was determined after a second round of binding and release. Each sample was tested in triplicate.

VEGF165 was analyzed in a cell proliferation assay using human umbilical vein endothelial cells (HUVECs) from SBH Sciences (SBH Sciences, Natick, USA). The HUVECs were incubated in different concentrations of VEGF165 for 88 h. Cell proliferation was then analyzed by measuring the difference in optical density at 490 nm. Each sample was tested in duplicate for each version of VEGF165 (VEGF165 commercial standard from ORF Genetics, and SES_VEGF165 produced in *N. benthamiana*).

### Analysis of plant SES performance in *S. cerevisiae*

The *S. cerevisiae* strain CEN.PK111-32D (*URA3*, *HIS3*, *leu2-3-112*, *TRP1*, *MAL2-8c*, *SUC2*) was used as a host to test the SES_B64_A system. The SES_B64_A and modified SES_B64_A—lacking the sTF expression cassette (negative control)—were transferred to a yest genome integration construct allowing the replacement of a non-functional *LEU2* allele in the CEN.PK111-32D strain. The original SES_A* version for *S. cerevisiae*, the corresponding negative control version (Rantasalo et al. 2018a), and the mCherry construct driven by the *PGK1* promoter were used for comparison. The linearized constructs were introduced into strain CEN.PK111-32D using the lithium acetate method. The resulting colonies were tested for single-copy genomic integration by RT-PCR using genomic DNA as a template and the primers listed in Table S4. Positive clones were cultivated in 24-well plates in YPD medium. The fluorescence of the yeast cell suspensions was measured as previously described (Rantasalo et al. 2018a).

### Statistical analysis

Shapiro-Wilk test was used to check for normal distribution. Significance in pairwise comparisons was determined using one way ANOVA with Tukey post-hoc test and Holm-Bonferroni comparison with SPSS Statistics for Windows v28.0 (IBM, Armonk, USA).

## Results

### Selection of the plant core promoters

The original SES showed unprecedented functionality in a taxonomically diverse range of fungi (Rantasalo et al. 2018b) suggesting it might also function in even more evolutionary distant organisms, such as plants. The original fungal SES did not yield detectable reporter expression in our transient expression assay in *Nicotiana benthamiana* (data not shown). To enable SES function in plants, we decided to implement CPs originating from plants into the SES system, while keeping the other components intact. The model plant *Arabidopsis thaliana* was selected as a source organism for the CPs, due to the wealth of published transcription data, including information about 5’-UTRs. The candidate *A. thaliana* CPs were selected from promoters of genes showing high expression levels (Wickramasuriya and Dunwell 2015), indicating a good capacity of CPs to efficiently recruit the transcription machinery for triggering strong transcription. The same selection criteria were applied, as used in establishing the original SES, namely by identifying the presence of a TATA-like sequence ~ 150 bp upstream of the start codon (Table S1).

Fifteen candidate *A. thaliana* CPs (Table S1) were tested by co-transforming them into *S. cerevisiae* with centromeric, linearized plasmid and pooled colonies for each transformation were tested for mCherry fluorescence with a fluorometer according to the previously established CP screen in *S. cerevisiae* (Rantasalo et al. 2018a) to identify those with features allowing efficient transcription even in fungi, indicating a universal function in diverse eukaryotes (Fig. 1a). CPs with various levels of functionality were identified in yeast based on the mCherry fluorescence signal (Fig. 1b). Five candidates were then used to optimize SES for plants.

### Establishment of the plant-optimized SES

Individual components of the original fungal SES were adapted to better match the gene expression requirements in *N. benthamiana* (Fig. 2a). First, the sTF and mCherry open reading frames were codon optimized for plants (Table S2). The sTF fusion protein was composed of a DNA-binding protein of prokaryotic origin (Bm3R1 or TetR) that previously showed excellent performance in the fungal SES (Rantasalo et al. 2018b), and the SV40 nuclear localization signal, coupled to a transcription activation domain (AD) based on either a single copy of VP16, or four tandem copies, known as VP64 (Beerli et al. 1998; Lowder et al. 2018). Transcriptional terminators for the mCherry and sTF genes were selected from strongly expressed *A. thaliana* genes with short 3′-UTRs (Table S2). The five *A. thaliana* CPs were then introduced into the original SES, favoring the use of At-TCTP1cp to express the sTF, resulting in seven different versions of the plant SES (Fig. 2a).

To test the functionality of plant SES components *in planta*, each construct and a corresponding negative control lacking the sTF expression cassette were cloned into a SES modified version of vector pJJJ178 (Reuter et al. 2016). In transient expression assays, leaves infiltrated with the full SES constructs showed substantially higher mCherry fluorescence intensity compared to no-sTF negative controls confirming sTF-dependent activation of the promoter (Fig. 2b). The latter presumably indicate minimal mCherry transcription caused by the basal activity of the CP (cp. #1 in Fig. 2a) in the absence of the sTF, or (in the cases of SES_T64-A and SES_T16-A) low intrinsic transcriptional activation of the synthetic mCherry promoter. Bm3R1-based sTF construct variants yielded higher fluorescence compared to the TetR based counterparts, indicating better function and performance in the *N. benthamiana* assay. Interestingly, the enhanced version of the VP64 activation domain resulted in no appreciable increase of fluorescence signal relative to VP16. Finally, the construct which appeared to be the most effective (SES_B64-A), was chosen for subsequent experiments.

### Expression level tuning using SES

The precise control of heterologous gene expression in plants is challenging (Buyel et al. 2013). To enable precision expression in *N. benthamiana*, the basic SES_B64-A, with eight sTF biding sites in the mCherry synthetic promoter, was modified to contain 0–16 binding sites (Fig. 2c). Additionally, an expression construct with a conventional structure was generated, using the well-established double-enhanced CaMV 35 S promoter (2 × 35 S) (Odell et al. 1985) as a control (Fig. 2d). The activity of SES and control constructs was assessed by transient expression in *N. benthamiana* plants. The number of binding sites correlated closely with the intensity of mCherry fluorescence, and brown/red pigmentation was visually observed in the infiltrated leaves of SES constructs with the greatest number of binding sites (Fig. 3a). The visual observation was quantified by fluorimetry in leaf extracts 6 days post-infiltration (Fig. 3b). The fluorescence intensity increased according to the number of binding sites, reaching a plateau with eight. The mCherry fluorescence elicited by the control construct featuring the 2 × 35 S promoter was markedly lower than that obtained with SES constructs containing 8–16 binding sites, being slightly lower than that of the four binding site construct.

The qRT-PCR analysis was conducted on cDNA samples produced from mRNA isolated from leaves 6 days post-infiltration, (Fig. 3c and S2) and confirmed that mCherry transcript levels were interdependent on the number of binding sites but also revealed additional phenomena. The relative expression of mCherry in SES constructs with 8–16 binding sites, normalized to UBC-e2, significantly exceeded that of the RBCS gene, one the most active native genes in plants (Tabita et al. 2007). Another observation was the low relative expression of mCherry driven by the 2 × 35 S promoter 6 days post-infiltration (Fig. 3c), which was below the levels of mCherry expressed with SES featuring a single sTF binding site. This appears to contradict the fluorescence intensity in the same samples, where the fluorescence of mCherry driven by the 2 × 35 S promoter was closer to that of the SES construct with four binding sites (Fig. 3b). This may reflect the specific characteristics of transcriptional regulation by the 2 × 35 S promoter, prompting to further investigate its dynamic expression profile.

### Constitutive SES functionality independent of p19

**Fig. 4:**
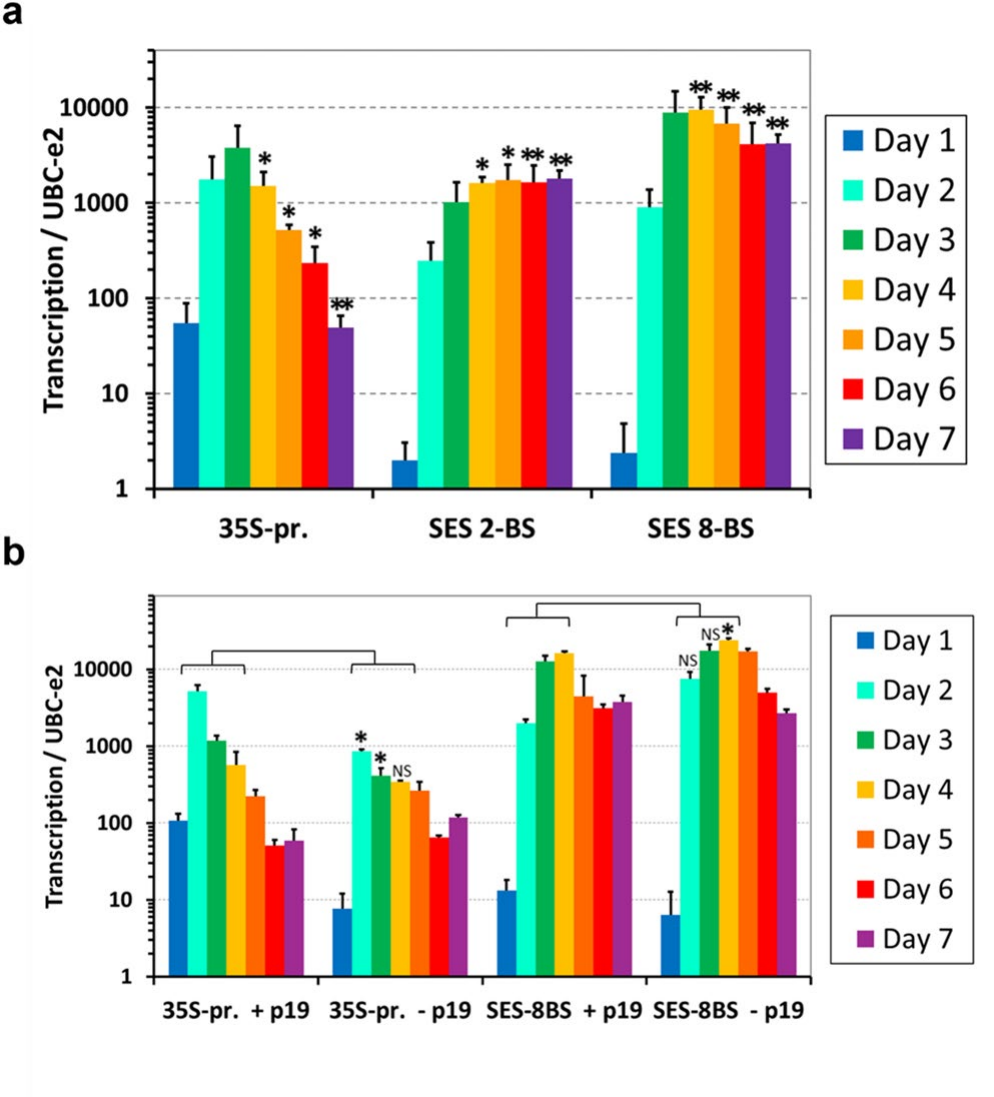
**a** Time-course analysis of mCherry mRNA in selected constructs. The expression of mCherry in *N. benthamiana* leaves infiltrated by *R. radiobacter* carrying either a 2 × 35 S promoter construct, (Fig. 2d) or a specific version of the SES (two or eight binding sites, Fig. 2c), combined with the p19 expression cassette. Expression profiles of mCherry mRNA in leaves collected 1–7 days post-infiltration were compared to the constitutive *UBC-2e* gene. The values are plotted in a log₁₀ scale, Data are means + standard deviations of two biological replicates (two different plants) and at least four technical replicates. Statistically significant differences were determined by applying a two-tailed Student’s *t*-test comparing the highest day expression (day 3) of each construct to the consecutive days (*P < 0.05, **P < 0.01). **b** Expression of mCherry in *N. benthamiana* leaves infiltrated by *R. radiobacter* carrying either a 2 × 35 S promoter construct, or eight binding site version of SES comparing the transcription efficiency of 2 × 35 S (Fig. 2d) and SES-8BS promoters (Fig. 2c) with and without p19. Leaf samples for mRNA were collected 1–7 days post-infiltration and were compared to the constitutive *UBC-2e* gene. The values are plotted in a log₁₀ scale, Data are means + standard deviations of two biological replicates (two different plants) and at least four technical replicates. Statistically significant differences were determined by applying a two-tailed Student’s *t*-test comparing matched time points between with‑p19 and without‑p19 conditions (*P < 0.05, NS = no significant difference)

Levels of mCherry mRNA were measured during a 7-day time course in *N. benthamiana* leaves infiltrated with SES versions containing two or eight binding sites, compared to the 2 × 35 S promoter system co-transformed with Tombusvirus p19 silencing suppressor (Garabagi et al. 2012; Jay et al. 2023) (Fig. 4a). For all three systems, the maximum level of mCherry expression was detected 3 days post-infiltration. The 2 × 35 S promoter was associated with a faster onset of mCherry expression at day 1 and 2 as compared to the SES versions, but steady state transcript levels began to wane by day 3 and declined to negligible levels by day 7. In contrast, mCherry mRNA levels driven by SES remained relatively stable, with low significant difference between day 3 and consecutive days (*p* < 0.5), although the levels declined in the system with eight binding sites after day 5 (Fig. 4a). There was a consistent difference in the relative expression of mCherry between the versions with two and eight binding sites, highlighting the ability of SES to maintain a consistent level of expression. The repeated 7-day time-course analysis showed a marked decrease in the mCherry transcript levels driven by the 2 × 35 S promoter in the absence of p19, while the expression of mCherry driven by the SES construct showed greater transcript stability over time. Notably, SES-driven expression without p19, showed somewhat higher mCherry transcript levels compared to conditions with p19 co-expression (Fig. 4b), demonstrating that SES-driven expression is both more stable and less dependent on silencing suppression than 2 × 35 S. The dynamic difference between the expression profiles of the 2 × 35 S promoter and SES implied that the two systems most likely affect the intrinsic gene silencing machinery in plants differently (Hamilton et al. 2002; Johansen and Carrington 2001; Tan et al. 2020).

### Production of heterologous proteins

**Fig. 5:**
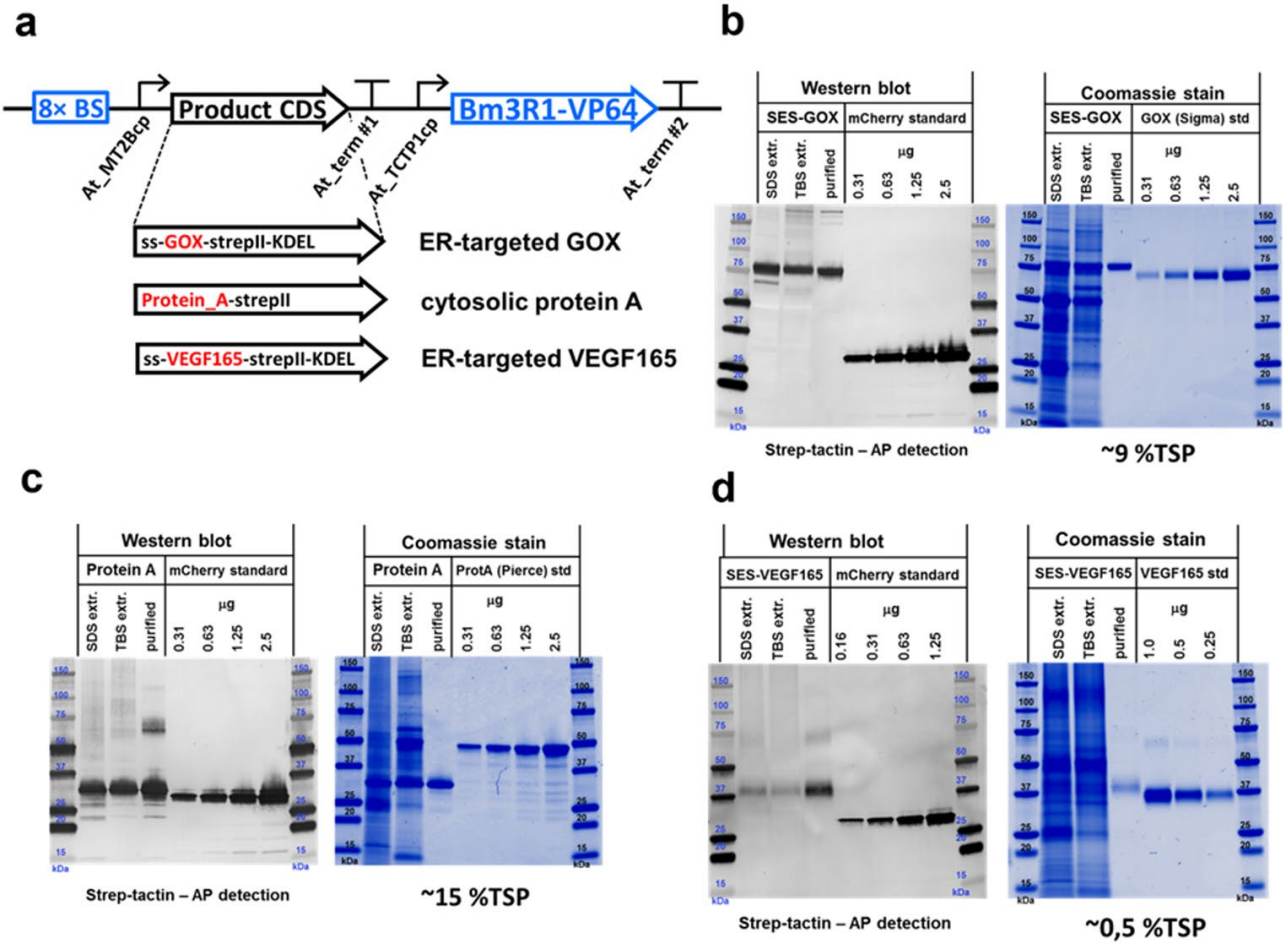
**a** Production of diverse candidate proteins in *N. benthamiana* using SES. Schematic representation of the SES constructs for the production of fungal glucose oxidase (GOX), bacterial protein A, and mammalian VEGF165. All constructs were based on version SES-B64-A (Fig. 2a), replacing the mCherry coding region with the corresponding sequence of each target protein, plus additional sequences to facilitate subcellular localization and purification. **b** Production of ER-targeted glucose oxidase (GOX). The apparent molecular weight (MW) of the purified protein was higher than that of the commercial GOX standard due to presence of the N-terminal signal sequence (SS), C-terminal STREP-II tag, and the ER-retention signal (KDEL). The product yield in the infiltrated leaves was ~ 1.0 g kg^−1^ FW. The predicted molecular mass of the SES-GOX is ~ 69 kDa. **c** Production of the N-terminal domain of protein A and its cytosolic accumulation. The product yield in the infiltrated leaves was ~ 1.4 g kg^−1^ FW. The predicted molecular mass of truncated protein A with the C-terminal STREP-II tag is ~ 34 kDa. **d** Production of ER-targeted VEGF165. SDS-PAGE under non-reducing conditions (without β-mercaptoethanol) was used to assess the production of disulfide-linked homodimers in *N. benthamiana*. The predicted molecular mass of the monomer is ~ 24 kDa, including the N-terminal signal sequence (SS), the C-terminal STREP-II tag, and the ER-retention signal (KDEL). The product yield in the infiltrated leaves was ~ 85 mg kg^−1^ FW. Proteins from leaf extracts were prepared in SDS buffer or TBS 6 days post-infiltration. The TBS extracts were used for affinity purification. The total protein extracts and the purified proteins were analyzed by western blot (left panel) or by Coomassie staining (right panel). Purified mCherry with a C-terminal STREP-II tag was used as a loading control in the western blot experiments. Commercially available GOX, protein A and VEGF165 were loaded as controls in the SDS-PAGE experiments. Precision Plus Protein Standards (Bio-Rad) were used as markers. The %TSP and absolute yields (g/kg FW) were calculated by densitometry (Fig. S5)

To assess the ability of SES to produce recombinant proteins in *N. benthamiana*, three commercially relevant proteins with diverse properties and origins were selected as targets. To enhance cloning efficiency, a high copy number *R. radiobacter* vector (pK2GW7) (Karimi et al. 2002) was used as a backbone for creating SES expression cassettes for protein expression (Fig. S1e–f). SES vectors with eight binding sites (based on *pK2GW7-SES_B64-A* ) were constructed by replacing the mCherry coding region with that of fungal GOX (Bankar et al. 2009; Wong et al. 2008), bacterial protein A (Deisenhofer 1981; Shukla et al. 2007), or human VEGF165 (Neufeld et al. 1994; Siemeister et al. 1998) (Fig. 5a). The proteins were allowed to accumulate in the cytosol, or an N-terminal Pr1b signal peptide and C-terminal KDEL retention signal (Joensuu et al. 2010; Kurppa et al. 2018) were added to direct proteins into the secretory pathway and ensure their accumulation in the endoplasmic reticulum (ER). All three proteins also contained a C-terminal STREP-II affinity tag to facilitate detection and purification. The correct subcellular localization was tested and confirmed by confocal microscopy using mCherry fusion proteins produced with the corresponding modifications in *N. benthamiana* (Fig. S3a–c).

*Aspergillus niger* GOX is an industrial enzyme with multiple uses in the food industry and other fields (Wong et al. 2008). The native enzyme contains a short N-terminal peptide that is removed during secretion, yielding a truncated extracellular product. For production in *N. benthamiana*, the native signal sequence was removed (cytosolic accumulation) or replaced with the tobacco Pr1b signal peptide (accumulation in the ER). Cytosolic GOX expression appeared to be highly toxic, resulting in necrotic lesions colocalized with the infiltrated areas of *N. benthamiana* leaves within 2 days post-infiltration, and no living tissue could be recovered for analysis. In contrast, ER targeting supported high GOX protein accumulation, with and estimated amount of ~ 1.0 g kg^−1^ fresh weight (FW), equivalent to ~ 9% TSP based on densitometry of SDS-PAGE and Western blot relative to standards (Fig. 5b; Fig. S5). GOX purified from *N. benthamiana* retained most of its specific activity as shown by comparison with commercially available GOX in an enzyme assay (Fig. S4a).

Protein A is a cell-surface protein from the pathogenic bacterium *Staphylococcus aureus*. It is well known for its ability to bind immunoglobulins and is used for product recovery during the industrial purification of antibodies. The native protein features a C-terminal domain that binds the cell wall and an N-terminal domain that binds immunoglobulins. Only the N-terminal domain in *N. benthamiana* was expressed, either in its native form for cytosolic accumulation (Fig. 5c) or targeted to the ER using the Pr1b signal peptide (not shown). Both approaches led to similar outcomes, and the accumulation of protein A did not cause any visible adverse effects. Protein A accumulation was estimated at ~ 1.4 g kg^−1^ FW, equivalent to ~ 15% TSP (Fig. 5c). Full functionality was verified by comparison with commercially available protein A in an IgG-binding assay (Fig. S4b).

VEGF165 is a regulatory protein with multiple roles in tissues repair, bone formation, cell proliferation and differentiation. It is a major splicing isoform of the human *VEGF-A* gene, producing a polypeptide 165 amino acids in length that forms a disulfide-linked homodimer (Eming and Krieg 2006). Production in *N. benthamiana* was only possible when targeting the ER because the cytosolic accumulation of VEGF165 caused localized tissue necrosis shortly after infiltration. The yield was also lower than the other two proteins at ~ 85 mg kg^−1^ FW, equivalent to ~ 0.5% TSP (Fig. 5d). Purified VEGF165 protein supported HUVEC proliferation, although at a lower efficiency compared to commercially available VEGF165 in cell proliferation assay (Fig. S4c).

### One expression system that functions in two distinct eukaryotic kingdoms

The successful development of SES for *N. benthamiana* relied on broadly functional core promoters from *A. thaliana*. Given that the CP screen was carried out in *S. cerevisiae*, it was hypothesized that the plant-optimized SES would also function in fungi. However, CPs alone are not sufficient for the full function of SES because the coding regions and sTFs in particular also need to match the codon preference of the host (Rantasalo et al. 2018a). The codon usage in *N. benthamiana* and *S. cerevisiae* was not compared in detail, but the GC content is similar: ~40% in *S. cerevisiae* (www.kazusa.or.jp/codon/) and ~ 42% in *N. benthamiana* (Kurotani et al. 2023).

**Fig. 6:**
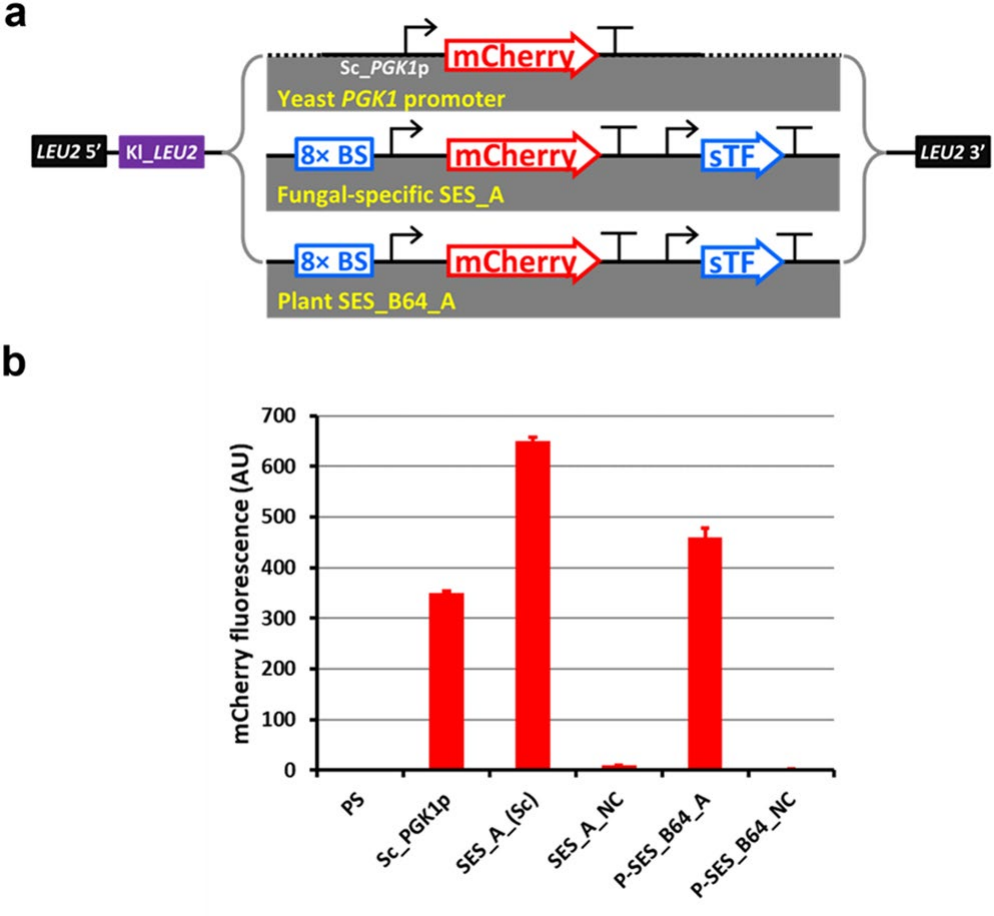
Functionality of SES across two eukaryotic kingdoms. SES_B64-A developed for *N. benthamiana* and the corresponding negative control (Fig. 2a) were transferred to a *S. cerevisiae* plasmid for single-copy genome integration. The original yeast-specific SES (Rantasalo et al. 2018a) and a construct with mCherry driven by the *S. cerevisiae PGK1* promoter were also integrated in *S. cerevisiae* as controls. **a** Schematic representation of the constructs used in *S. cerevisiae*. All constructs were inserted into the same plasmid backbone allowing genome integration into the *LEU2* locus. **b** Quantitation of mCherry fluorescence in *S. cerevisiae* strains normalized to the optical density of the cell suspensions. Data are means + standard deviations of at least *n* = 3 independent cultivations. PS—parental strain

To directly test the hypothesis, the SES_B64-A and the corresponding negative control was transferred (Fig. 2a) into a plasmid enabling single-copy integration into the *S. cerevisiae* genome at the *LEU2* locus. The production of mCherry was assessed in *S. cerevisiae* strains carrying SES_B64-A, the original fungal SES-A, or mCherry driven by the strong native *PGK1* promoter (Fig. 6a). The mCherry fluorescence in the strain carrying the SES_B64-A construct was significantly higher than that achieved with the *PGK1* promoter (Fig. 6b). This confirmed that SES_B64-A functions properly in *S. cerevisiae* and accordingly that the plant-optimized SES facilitates high-level heterologous gene expression in two distinct eukaryotic kingdoms.

## Discussion

This work describes the development of a new version of our synthetic expression system (SES) that allows the constitutive and tunable expression of heterologous genes in plants. The original SES was functional in many diverse fungal species, particularly ascomycetes (Rantasalo et al. 2018a). To extend the functionality to plants, exemplified here by *N. benthamiana*, SES was modified by codon optimization and by replacing the fungal core promoters (CPs) and transcriptional terminators with plant counterparts. Because all these modifications were introduced simultaneously, their individual contributions for the activity of the plant SES could not be assessed.

The selection of plant CPs was analogous to the fungal CP screening process (Rantasalo et al. 2018b), but the source organism was *A. thaliana* (Fig. 1). CPs representing strongly expressed genes (Wickramasuriya and Dunwell 2015) were manually inspected for the presence of a short 5′-UTR and upstream TATA-like box, thus narrowing down the number of candidates for assessment. The function of the preselected CPs was then evaluated in *S. cerevisiae* (Rantasalo et al. 2018a), which may limit the selection by excluding those active in plants but inactive in fungi. To optimize the plant SES even further, stronger plant-specific CPs could be screened directly in *N. benthamiana* and/or with more relaxed selection criteria. This process could be accelerated by the use of automated high-throughput methods, such as in silico algorithms for CP selection and/or robot-assisted cloning and screening.

The best candidate CPs in this study (At-MT2Bcp and At-TCTP1cp; Table S1) were used to construct the SES_B64-A variant (Fig. 2). At-MT2Bcp is derived from the *MT2B* promoter, which drives a gene encoding a small cysteine-rich metallothionein (Guo et al. 2008). At-TCTP1cp is derived from the translationally controlled tumor protein (TCTP1) promoter, which drives a gene involved in cell cycle regulation that is conserved in animals and plants (Branco and Masle 2019; Brioudes et al. 2010). This indicates that the CPs that are highly functional in SES, could originate from very diverse genes whose promoters are not typically considered suitable for high level heterologous gene expression.

Another key component of SES is the synthetic transcription factor (sTF), an artificial fusion protein composed of DNA-binding and transcriptional activation domains. Two DNA-binding proteins (Bm3R1 and TetR) were tested, which were previously found to be suitable for the fungal SES (Rantasalo et al. 2018b). TetR is used in the doxycycline-dependent TET-ON/OFF expression systems, which are functional in various types of organisms, including plants (Linesch et al. 2023; Zhou et al. 2017). However, the plant SES containing the TetR-based sTF showed an unexpectedly low level of mCherry production (Fig. 2b), and two negative control constructs showed a high level of background fluorescence in the absence of sTF, possibly due to the specific combination of the TetR-binding sites and At-MT2Bcp (Fig. 2a). Other explanations, such as intrinsic activation via the TetR binding site or At-MT2Bcp alone, were excluded based on the behavior of the constructs containing these DNA parts individually, and the lack of background activation (leakiness) when the TET-ON system was used in BY-2-cells (Bortesi et al. 2012; Nagata et al. 1992). Furthermore, the VP64 activation domain unexpectedly achieved only a marginal improvement over the activity of VP16 (Fig. 2b).

The functionality of the plant SES in *N. benthamiana* fulfilled our expectations, especially regarding its two most critical functional characteristics (Fig. 3). First, it allowed tunable expression based on the number of sTF binding sites in the target gene promoter, and second it significantly exceeded the mRNA levels produced using the CaMV 2 × 35 S promoter (Fig. 3c). In addition, insights into the function of the plant SES were obtained by analyzing the mRNA levels of the candidate genes in the infiltrated *N. benthamiana* leaves. Maximum expression levels were achieved when using eight sTF binding sites—more sites did not increase expression levels any further. This may reflect the limiting amount of sTF produced by SES, thus preventing a higher degree of occupancy in SES versions with 12 and 16 binding sites. Alternatively, the bottleneck could be a limited supply of native transcriptional machinery components, which would set a maximum rate of transcriptional initiation at the At-MT2Bcp. A low level of sTF transcription was expected and observed (Fig. S2) because the sTF gene was driven solely by the CP (cp. #2 in Fig. 2a). However, sTF expression was noticeably higher in the SES versions with 8–16 binding sites (S2). This may reflect the phenomenon of distant transcriptional activation (Schmitz et al. 2022), where the large number of sTFs recruited on the target gene promoter partially enhances the transcription of a downstream CP driving the sTF (Fig. 2c).

SES constructs with 8–16 binding sites revealed a phenomenon related to the downregulation of RBCS expression. This may partially be associated with large amounts of mCherry protein altering physiological responses in leaves and limiting the expression of the light-dependent RuBisCO small subunit gene. However, absorbance of mCherry overlaps only on the green to orange spectrum (450–590 nm) of visible light, so a more robust explanation would be that the photosystem is inhibited by pathogen related immune responses, which have been shown to downregulate RuBisCo, and possibly due to metabolic overburden related to high abundance of proteases secreted due to heterologously expressed proteins (Grosse-Holz et al. 2018; Robert et al. 2015). We did not systematically assess underlying physiological mechanisms regarding immune responses or metabolic burden in *N. benthamiana* but this could be a worthwhile target of future work.

Important observations were made when performing the time-course analysis, comparing SES with the 2 × 35 S promoter system (Fig. 4). The obviously faster onset of mCherry expression driven by the 2 × 35 S promoter, one of the strongest known promoters, may reflect the direct mechanism of transcriptional initiation, in contrast, the slower activation of SES-driven expression is most likely caused by the need to accumulate a threshold amount of sTF before full activation of the sTF-dependent promoter. This slow initial activation of transcription might also be a reason for the slower and more gradual silencing of mCherry expression. Both systems reached maximum activity 3 days post-infiltration, after which the expression of mCherry driven by the 2 × 35 S promoter became significantly lower, possibly due to the activation of post transcriptional gene silencing (PTGS) triggered by the fast accumulation of mCherry transcript levels. This occurred despite the presence of the p19 silencing repressor, which is routinely used to improve heterologous gene expression in plants (Garabagi et al. 2012; Jay et al. 2023). Transiently expressed GFP driven by 2 × 35 S has been shown to trigger siRNA accumulation as early as two days after infiltration in *N. benthamiana* (Johansen and Carrington 2001; Kościańska et al. 2005), suggesting that strong promoter activity can rapidly induce PTGS, either inherently due to high levels of transgene RNA, or due to high proportions of aberrant mRNA transcripts (Jay et al. 2023). Initial trials showed that SES achieved moderately stable expression without p19 (Fig. 4b), with high mRNA accumulation at days 6 and 7 post-infiltration. The expression driven by SES with eight binding sites at the end of the time-course was over ten times higher than that of 2 × 35 S, and as high as the maximum activity of the 2 × 35 S promoter on day 3 (Fig. 4a), clearly highlighting a marked difference in expression dynamics between SES and the 2 × 35 S system. Plant expression systems that do not require p19 for optimal performance benefit from a simpler design, more predictable activity, and conservation of the resources that would otherwise be diverted to p19 expression (Garabagi et al. 2012). Activity in the absence of p19 has also been observed for some plant promoters used for heterologous gene expression in *N. benthamiana*, including the *Chrysanthemum morifolium* RBCS promoter, which has been used to express the monoclonal antibody trastuzumab (Garabagi et al. 2012).

The four diverse proteins that were transiently expressed in *N. benthamiana* demonstrate the versatility of SES for the production of recombinant proteins (Fig. 5, S4 and S6). High yields of fully functional GOX and protein A, were produced in amounts comparable to a previously reported approach using hydrophobin fusions to facilitate protein purification (Joensuu et al. 2010; Reuter et al. 2016), and higher than in a report on stably expressed protein A in *Nicotiana tabacum* plastids (Owens et al. 2024). The production of VEGF165 was more challenging and only a small amount of partially active protein was obtained, however, expressing VEGF165 in *N benthamiana* has been shown to be difficult, with protein production levels at much lower levels than in this study (Bulaon et al. 2020). GOX and VEGF165 caused localized tissue necrosis when accumulated in the cytosol, which in the case of GOX can be explained by the cytosolic production of gluconic acid and hydrogen peroxide from glucose causing oxidative stress to the infiltrated plant. The protein amounts of mCherry were not quantified, but it seemed substantially higher than that of ~ 1.4 g kg^−1^ protein A, when expressed in cytosol (Fig. S6). Modern expression vectors utilizing viral replicating systems have reported similarly high reporter protein amounts in transient expression experiments ranging from 30% tsp and 1 g kg^−1^ for pEff vector utilizing a potato virus X based system (Mardanova et al. 2017), to 5,5 g kg^−1^ for TRBO vector utilizing a tobacco mosaic virus based system (Lindbo 2007). These examples provide useful comparisons but rely on fundamentally different expression mechanisms based on viral replication.

In addition to protein production, SES could be useful for metabolic engineering because it would allow enzymes to be expressed at defined levels rather than defaulting to the maximum possible, which would be necessary for enzyme stoichiometry in some pathways. Establishing the plant SES as a transient expression system is also useful because it allows the rapid assessment of different constructs for the expression of proteins or the production of metabolites and could be used industrially to fulfil niche markets with fluctuating demand. Plant SES delivers orthogonal expression without the need of viral replication elements, negating problems with biosafety and downstream processing of viral-derived impurities.

At present, SES has only been validated in transient expression assays, and its performance in stable transgenic plants remains to be determined and will be the focus of future work.

Finally, we have demonstrated that the plant SES is also active in a fungal host, the yeast *S. cerevisiae* (Fig. 6). To our knowledge, this is among the first successful examples of an expression system that is functional in different eukaryotic kingdoms. The SES_B64-A variant was used in yeast without any sequence modification, yet the mCherry expression level significantly exceeded that achieved when using the strong native *PGK1* promoter. The establishment of a universal expression system that works across kingdoms could provide new opportunities in biotechnology, including the faster and more cost-efficient development of recombinant protein production or metabolic engineering strategies, due to the option of rapidly screening and assessing diverse hosts in parallel for optimal product formation.

## Electronic Supplementary Material

Below is the link to the electronic supplementary material.

Supplementary Material 1

## Data Availability

All data supporting the findings of this study are available in the supplementary material.
