# Supplementary material for: A synthetic expression system for orthogonal gene expression in Nicotiana benthamiana
Source: Plant Mol Biol. 2026 Jun 2;116(3):62. doi: 10.1007/s11103-026-01725-7 (PMC13230335; doi:10.1007/s11103-026-01725-7)
Supplement: Supplementary file 1 — Supplementary Material 1 [file 11103_2026_1725_MOESM1_ESM.docx]

A synthetic expression system for orthogonal gene expression in *Nicotiana benthamiana*

Plant Molecular Biology

Dominik Mojzita^1^, Anssi Rantasalo^2^, Markus Laurel, Hannu Hotti, Kirsi-Marja Oksman-Caldentey, and Heiko Rischer^1^

VTT Technical Research Centre of Finland Ltd., P.O. Box 1000, FI-02044 VTT, Espoo, Finland

^1^To whom correspondence may be addressed.
Email: [dominik.mojzita@vtt.fi](mailto:dominik.mojzita@vtt.fi) or [heiko.rischer@vtt.fi](mailto:heiko.rischer@vtt.fi)

^2^Currently at EniferBio Oy, Espoo, Finland.

# Supplementary figures:

**Figure S1: Cloning procedures for the plant synthetic expression system**

**Figure S2: Logarithmic-scale plot of the data shown in Figure 3c**

**Figure S3: Subcellular localization of mCherry in the infiltrated leaves**

**Figure S4: Functional analysis of three diverse proteins produced in *N. benthamiana* using the plant SES**

**Figure S5: Quantification of proteins produced using the plant SES based on densitometry**

**Figure S6: Total protein extraction comparing accumulation of mCherry and protein A in infiltrated leaves**

# Supplementary tables:

**Table S1: Candidate core promoters screened in *Saccharomyces cerevisiae***

**Table S2: DNA sequences used to construct the expression cassettes**

**Table S3: Primers used to construct the expression cassettes**

**Table S4: Primers and gene targets used for transcript analysis**

**Supplementary figures**

**Fig. S1**
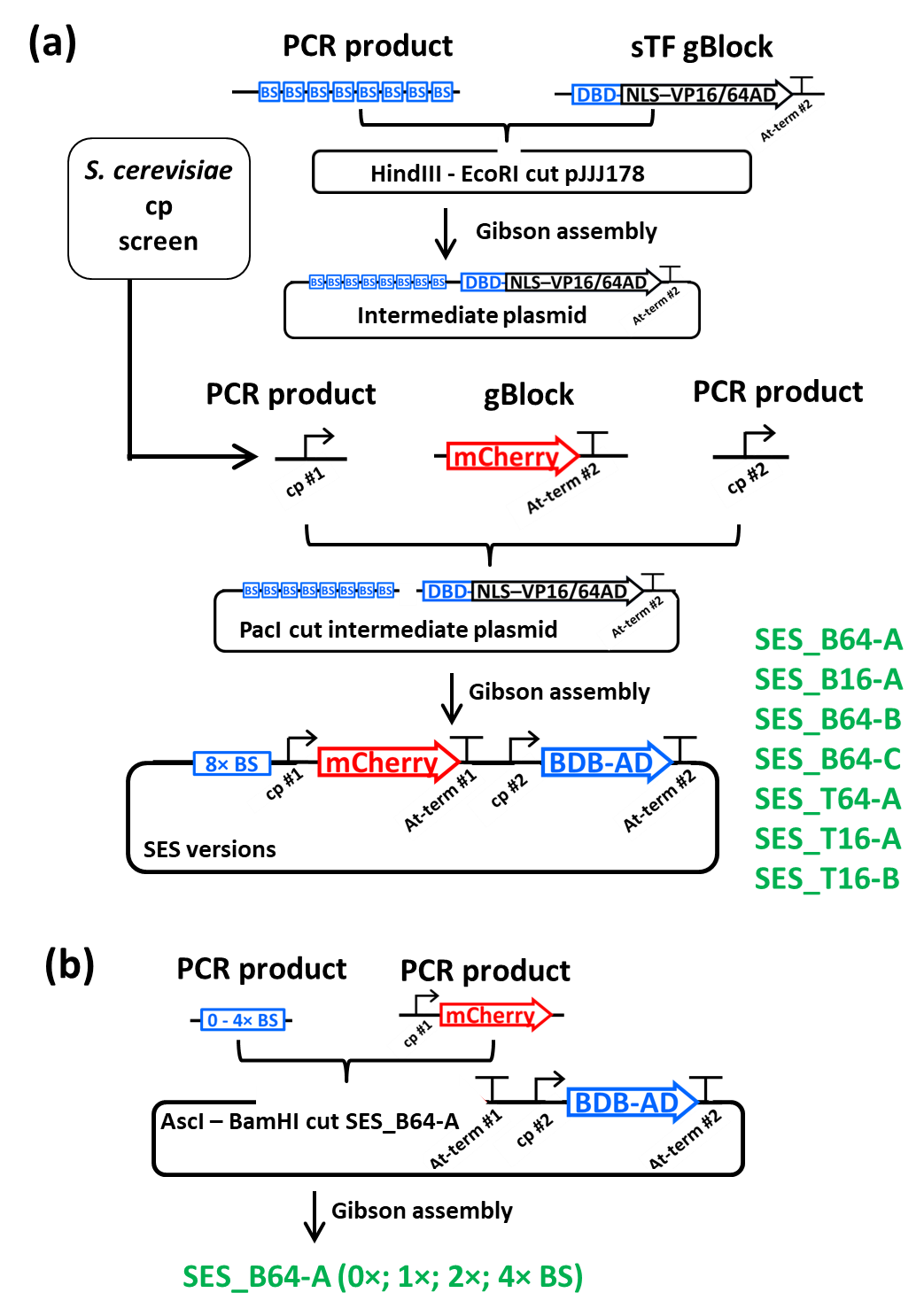


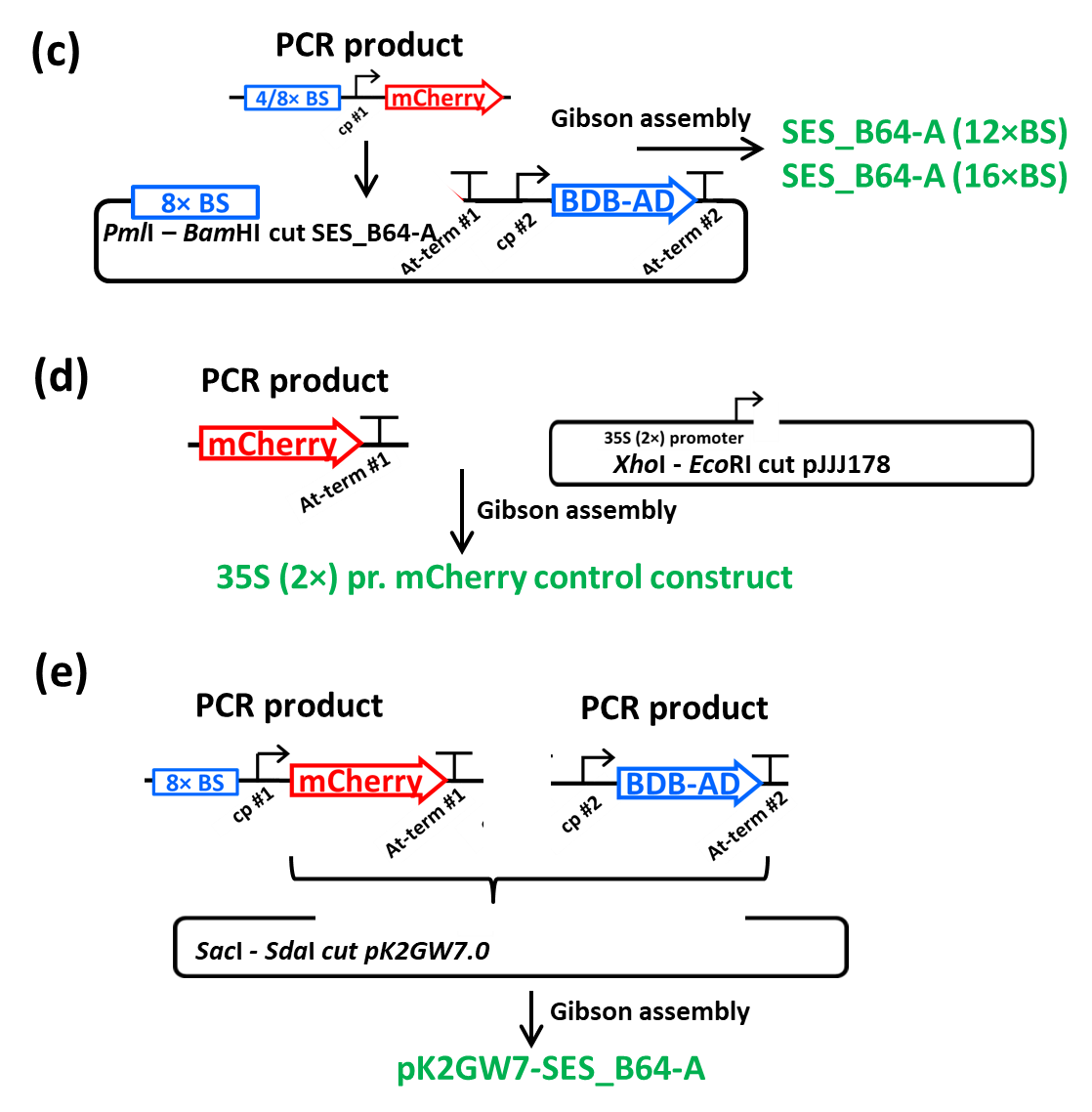


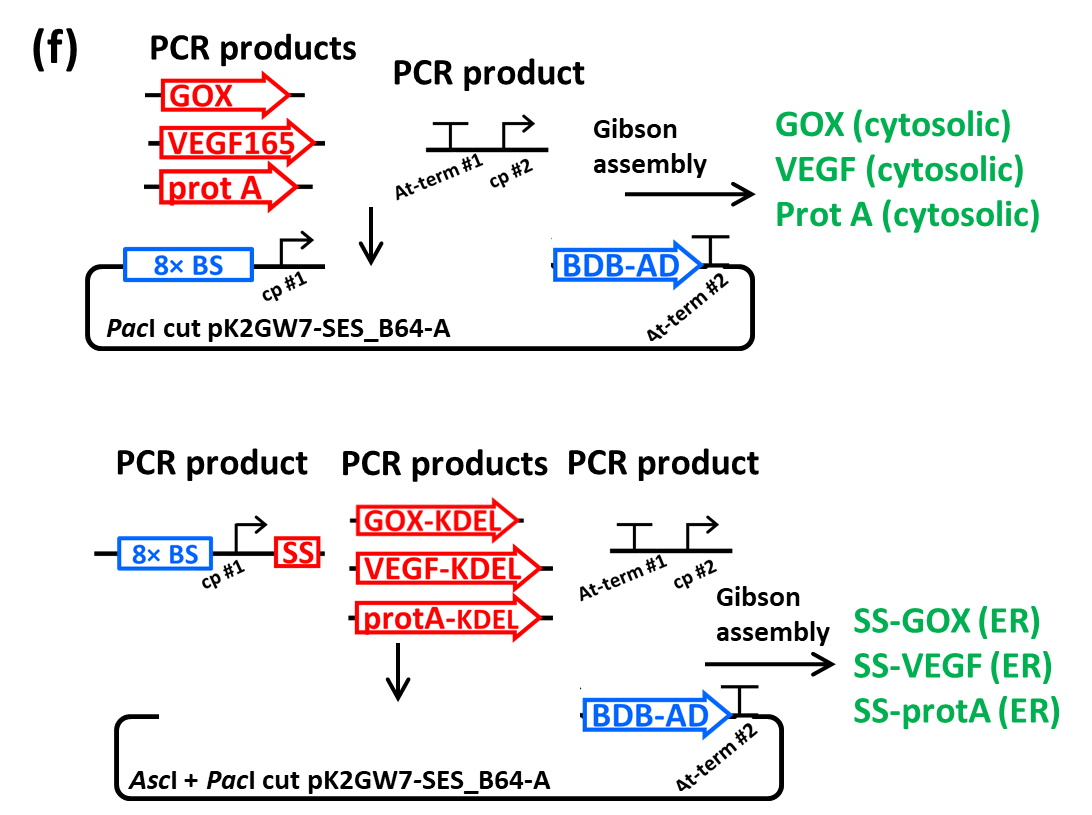


**Figure S1: Cloning procedures for the plant synthetic expression system**

The primers used to obtain the DNA fragments used in the constructs are listed in Table S3.

1. **Construction of the test SES versions shown in Figure 2a**. First, intermediate plasmids were constructed containing eight binding sites for sTFs based on Bm3R1 or TetR, and the corresponding sTFs with either VP16 or VP64 activation domains. The DNA inserts were amplified by PCR from existing plasmids (the construct with eight binding sites) or obtained as synthetic DNA (sTF gBlocks). The constructs were based on vector pJJJ178 (Reuter *et al.*, 2016). Second, the mCherry coding region and terminator were inserted into the intermediate plasmids, flanked by an upstream core promoter (forming the SES promoter for mCherry) and a downstream core promoter used to express the sTF. The core promoters were amplified from the constructs used in the core promoter screen in *S. cerevisiae*. The mCherry coding region was codon optimized for expression in *N. benthamiana* and obtained as a synthetic DNA (gBlock).
2. **Construction of SES_B64-A (Figure 2c) with 0–4 binding sites.** The SES_B64-A construct containing eight binding sites for Bm3R1 was modified by excising the mCherry expression cassette and reconstructing the expression cassette with fewer sTF-binding sites.
3. **Construction of SES_B64-A (Figure 2c) with 12 or 16 binding sites.** The SES_B64-A construct containing eight binding sites for Bm3R1 was modified by excising the mCherry expression cassette and reconstructing the expression cassette with additional sTF-binding sites.
4. **Construction of the 2×35S (CaMV) promoter control construct (Figure 2c).** Vector pJJJ178 (Reuter *et al.*, 2016) – containing the 2×35S-promoter – was modified by inserting the mCherry coding region and terminator used in all SES versions.
5. **Transfer of the SES_B64-A system into the pK2GW7.0 plasmid.** The pK2GW7.0 plasmid was digested with *Sac*I and *Sda*I to remove the 35S promoter-based expression cassette with CmR-ccdB. Two PCR products obtained from the SES_B64-A construct containing eight binding sites were inserted into the pK2GW7.0 vector, replacing the original expression cassette creating a new base plasmid pK2GW7-SES_B64-A.
6. **Construction of SES-based expression cassettes for the production of GOX, protein A and VEGF165 in *N. benthamiana* (Figure 5a).** For all three proteins, expression cassettes were constructed allowing cytosolic or ER localization. The pK2GW7-SES_B64-A was used for all constructs. For cytosolic localization, the mCherry gene of SES_B64-A was replaced by the PCR-amplified coding region of GOX (template pmil001) (Joensuu *et al.*, 2010), VEGF (template – synthetic DNA), or protein A (template pJJJ531) (Kurppa *et al.*, 2018). For ER localization, the proteins were augmented with an N-terminal Pr1b signal sequence (SS) and a C-terminal KDEL tag.

**Fig. S2**

**Figure S2 Relative expression of sTF**

Analysis of relative expression of sTF in leaves collected 6 days post-infiltration. The relative gene expression levels of sTF was compared to the 0BS construct, having basal core promoter activity. The constitutive *UBC-2e* gene was used as a reference gene. Data are means + standard deviations representing at least two biological (four technical) replicates.

**Fig. S3**


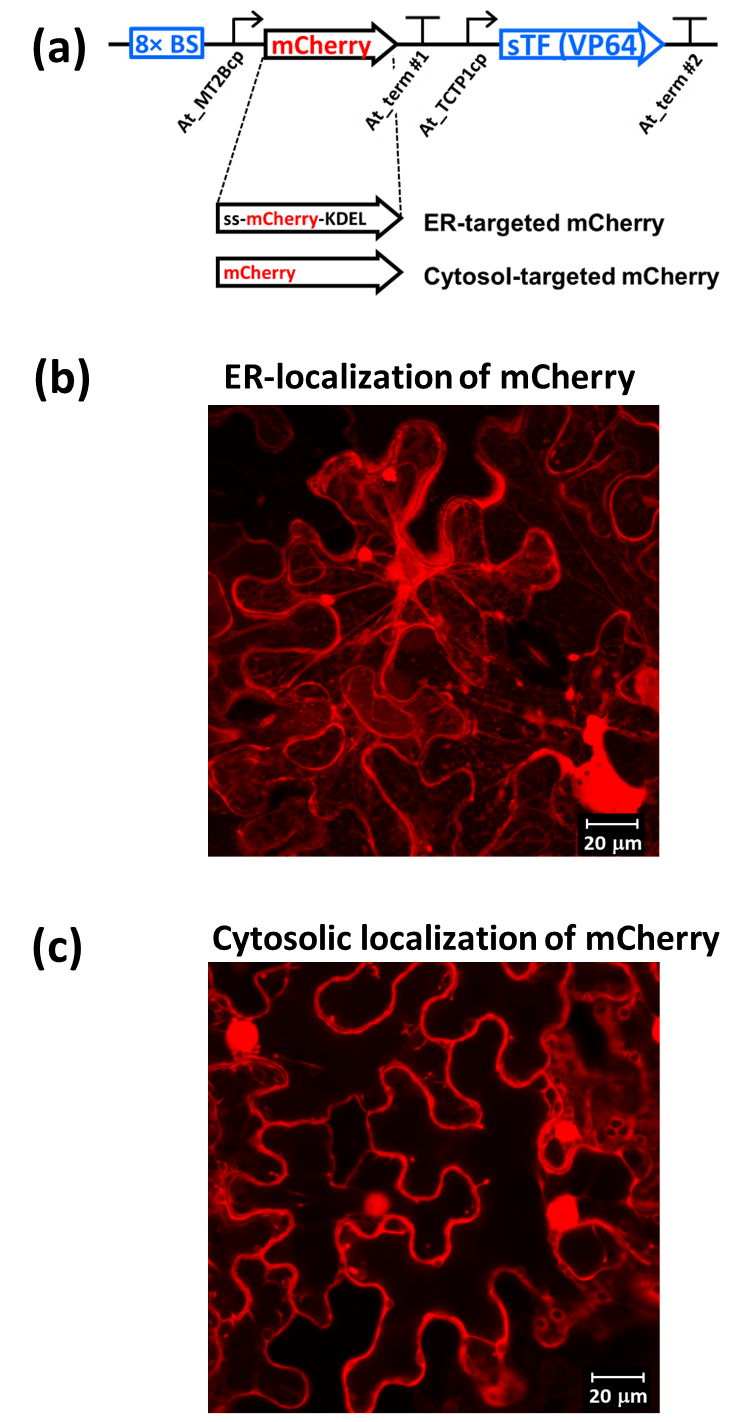


**Figure S3: Subcellular localization of mCherry in the infiltrated leaves**

Localization of mCherry was visualized using a Zeiss LSM 710 laser scanning confocal microscope (Carl Zeiss, Oberkochen) with a 63× water immersion objective (excitation at 543 nm and detection at 586–670 nm).

1. Schematic representations of the SES constructs (based on version SES_B64-A) for the targeting of mCherry to the cytosol and ER.
2. Confocal image (red fluorescence) of a leaf section 5 days post-infiltration with the construct for ER-localized mCherry.
3. Confocal image (red fluorescence) of a leaf section 5 days post-infiltration with the construct for cytosolic mCherry.

**Fig. S4**

**
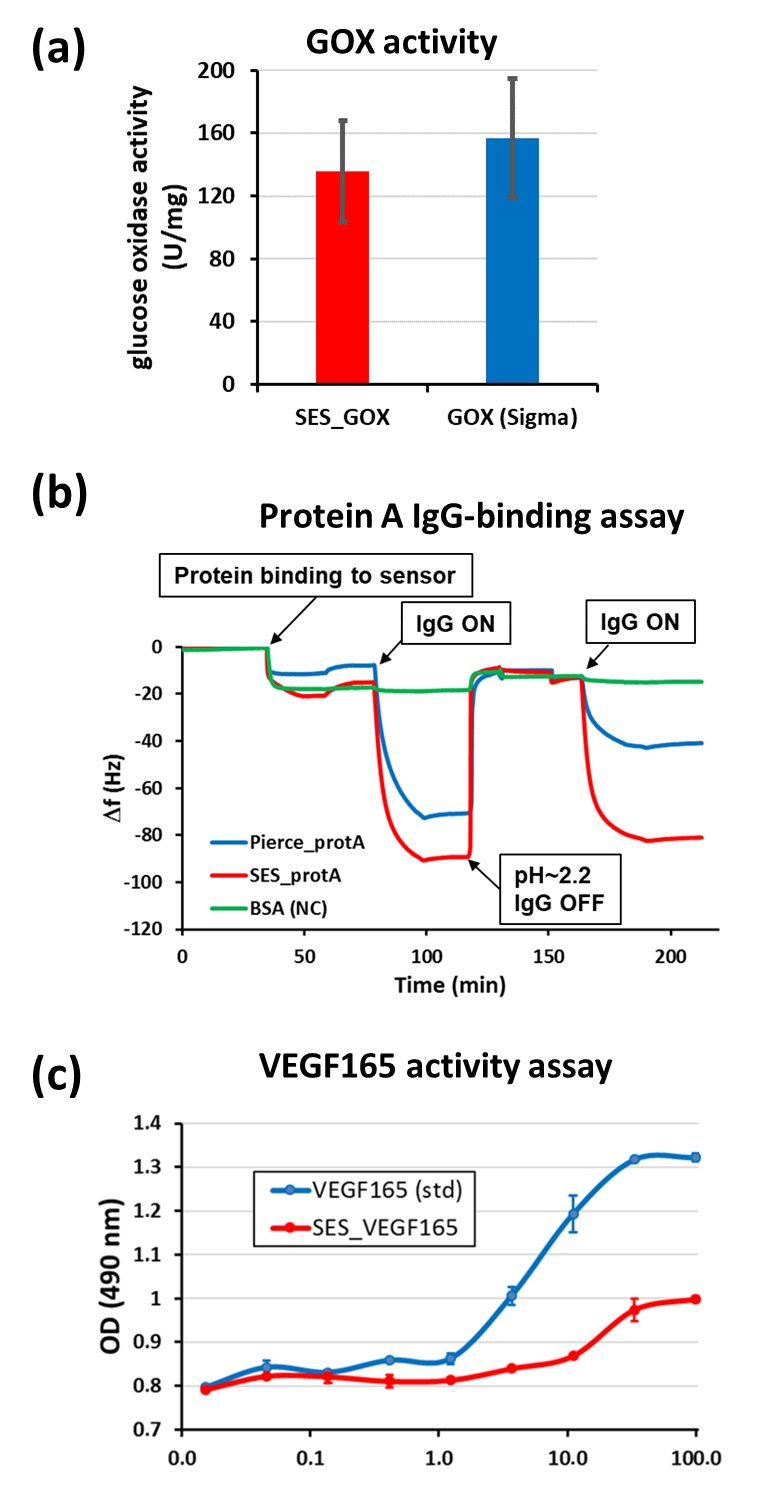
**

**Figure S4 Functional analysis of three diverse proteins produced in *N. benthamiana* using the plant SES**

Proteins purified from TBS leaf extracts (**Figure 5**) were tested using specific activity tests in comparison with commercially available standards.

1. The glucose oxidase produced in *N. benthamiana* (SES-GOX) and a commercial *Aspergillus niger* GOX obtained from Sigma-Aldrich (GOX-Sigma) were compared using a glucose oxidase assay kit (Megazyme). One unit of GOX represents the oxidation of 1 μmol d‑glucose in 1 min.
2. The protein A (antibody binding domain) produced in *N. benthamiana* (SES-protA) was compared to a full-length protein A obtained from Pierce (Pierce-protA) and bovine serum albumin (BSA) as a negative control (NC) for the ability to bind immunoglobulin G (IgG) in a real-time surface-molecule interaction experiment (Qsense). Following IgG binding, the sensor was transiently exposed to a low-pH (~2.2) buffer to release the bound IgG. A second round of IgG binding was tested after re-equilibration of the sensor with a wash buffer.
3. The VEGF165 produced in *N. benthamiana* (SES-VEGF165) and the recombinant human VEGF165 obtained from ORF Genetics (VEGF165 std) were tested in a proliferation assay using human umbilical vein endothelial cells (HUVECs) from SBH Sciences.

## Fig. S5

**(a)**

**GOX quantification – western blot**


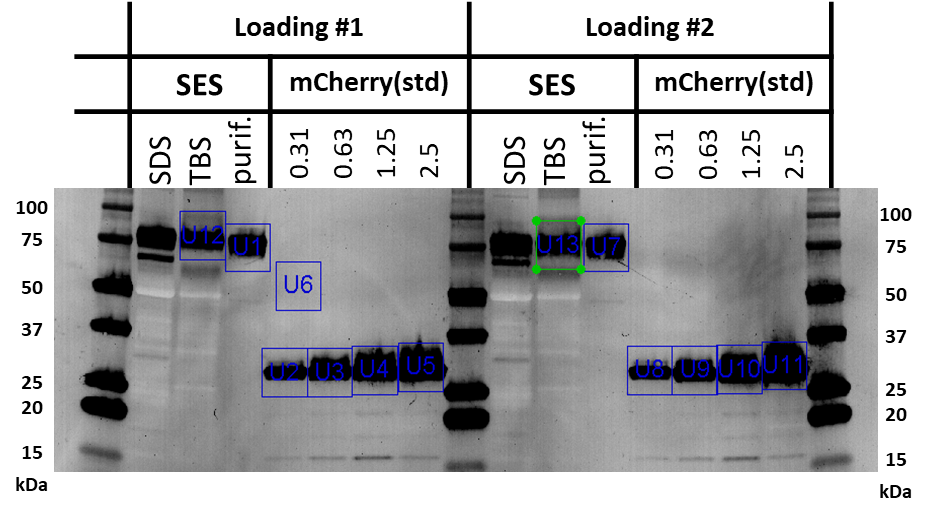


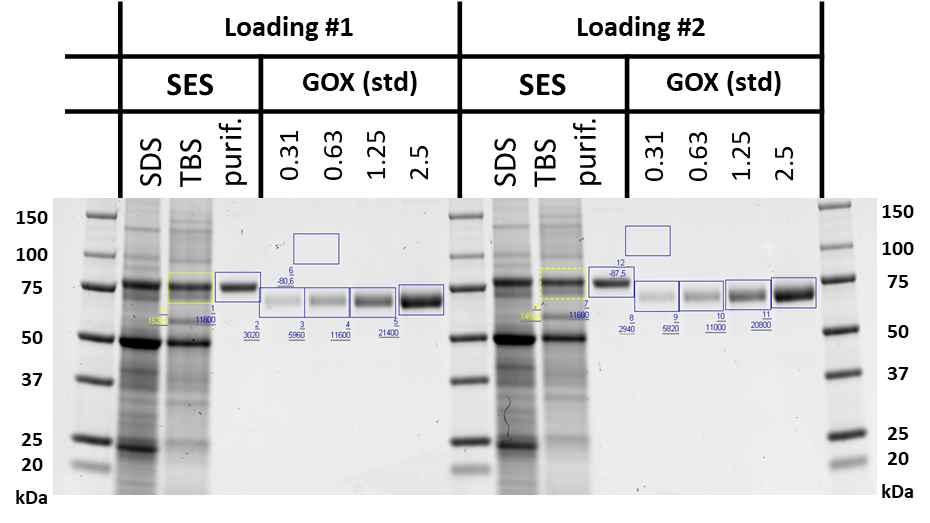


**GOX quantification - Coomassie**

**(b)**

1. **Figure S5: Quantification of proteins produced using the plant SES based on densitometry**
2. The three diverse recombinant proteins produced in *N. benthamiana* using the plant SES were quantified by densitometry to determine the percentage of total soluble protein (%TSP) as well as the absolute concentration of each protein in leaves (g/kg fresh weight). The %TPS was calculated using TBS extracts and the absolute concentration was estimated from the SDS extracts. The quantification of glucose oxidase (GOX) is shown as an example.
3. Western blot analysis. The samples and quantification standards (purified mCherry with STREP-II tag) were loaded twice on the gel. The blots were scanned using a GS-710 calibrated imaging densitometer (BioRad). The bands, corresponding to the proteins of interest, and the background areas were marked and quantified using Quantity One software (BioRad). The calibration curve was calculated for the mCherry dilutions and used for the calculations.
4. Coomassie staining and gel band analysis. The samples and quantification standards (in this case the commercial GOX protein) were separated by PAGE and the gel was stained with Coomassie Brilliant Blue. The gel was scanned using the Odyssey CLx Imaging System (LI-COR Biosciences). The protein bands and the background areas were marked and quantified using Image Studio (LI-COR Biosciences). The calibration curve was calculated for the commercial GOX dilutions and used for the calculations.

**Fig. S6**


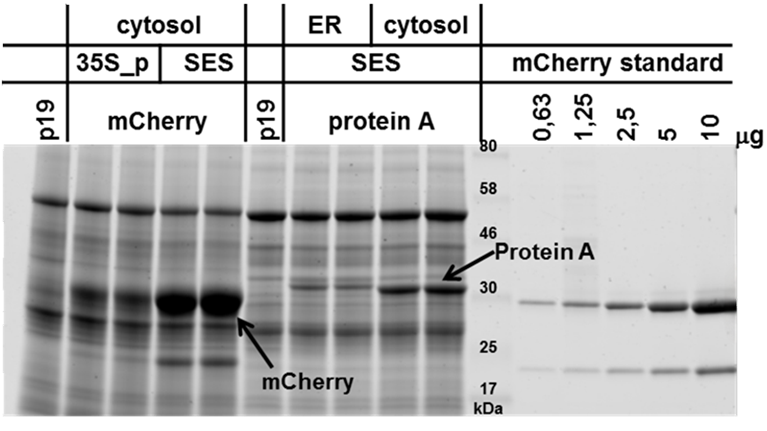


**Figure S6: Total protein extraction comparing accumulation of mCherry and protein A in infiltrated leaves**

Total SDS protein extraction of infiltrated *N. benthamiana* leaves. A comparison of 2x35S promoter and 8BS SES with protein A as a reference (~1,4 g kg⁻¹ tsp). The SDS-leaf extracts were separated by PAGE and the gel was stained with Coomassie Brilliant Blue. The gel was scanned using the Odyssey CLx Imaging System (LI-COR Biosciences).

# Supplementary tables

## Table S1

Candidate core promoters screened in *Saccharomyces cerevisiae* (**Figure 1**). The shaded sequences (9 bp) were added to the native core promoters (replacing the original 9 bp at the 3′-end) for screening and cloning purposes. The yellow shading indicates TATA-like sequences ~150 bp upstream of the start codon. The start codon (ATG) of the mCherry coding sequence is underlined. Sc-PGK1cp – *S. cerevisiae* core promoter used as a positive control; At – *Arabidopsis thaliana* core promoters. The source (transcript ID) of each Arabidopsis CPs can be retrieved from the genome database (https://phytozome-next.jgi.doe.gov/info/Athaliana_Araport11).

| **CP name** | **Gene source** | **Sequence (5′→3′)** |
| --- | --- | --- |
| Sc-PGK1cp |  | AAGGGGGTGGTTTAGTTTAGTAGAACCTCGTGAAACTTACATTTACATATATATAAACTTGCATAAATTGGTCAATGCAAGAAATACATATTTGGTCTTTTCTAATTCGTAGTTTTTCAAGTTCTTAGATGCTTTCTTTTTCTCTTTTTTACAGATCATCAAGGAAGTAATTATCTACTTTTTACAACAAATTAATTAAAATG |
| At-CRA1cp | AT5G44120 | AAGTCATAAATAGCAATTTAAGTGAAGTGTAAATTGTACATAGTCGACTCTATATACCTGGTTCTTATCTCATTCAATTTATCCTCAACAACTTTAATAGAAAAATATCAAATAAATTCCCTATAAATAGCTTCACATAATGCAAGTGAGAAACCACAAAAAGTAAGAAATATAAGATTAATTAAAATG |
| At-RPL41Dcp | AT3G08520 | ATCCCCTCTGGCAAATTCTTATCCATTTGGGTTTTATTGGGCTTTTGAAATAATAAAGCCCATTAAGTTAGTTACTAGGGTTTTGTTGTTGTTTAAAGGAGGAATAAGAGCGTAAGCTACAAAATCTTTCTATTCATCTCCGCCGCTCCTCATCCTGTAAAGCTAAACAAATAATCAGAGGAACGAAGGAGACAGCTTCTGCTTAATTAAAATG |
| At-ATTI7cp | AT1G47540 | GAATTTGTGGTTCTCGTGAAGTCGTGATAATAGTTTGTCCAAGCGATAAATATAAAATAGTATTGCACCTCAACAAGTGTTAAGCATGCAAATCCATTTACGCATACATATTAACTCCGAGTGAAATATAAATATTAGAGAGTAGAGACAGAGAAAAAGACAGAGACAAAGTTAATTAAAATG |
| At-THI1cp | AT5G54770 | ATCGTTACTTTCCATTGATGGCTAAAAATTAAAATAATCACGATAAATATTAATAATACAAAAAACAATTAAAATAACAAAAAAAGATCAAAAATTCTCTAACCCTTCATTCCTTATCTCTGACGTGGCCATCAATCTTCAGATTTTCTTCTTCTTCTAATTTAAATACTCAACAACCACTCTTCACTTCACCATCAGCATCACTAAACTCGAACCCTAAAGTTAATTAAAATG |
| At-MT2Bcp | AT5G02380 | GTGGACAAAGATCGTTGACACGTGGACGGTCTACAAATTCTAATTTTGCCTATAAATATCAAAGCTCCTGAATATGTAAGTTTCATTCACTGATTATCGTTTAAGGCAAATTAAGATCATCTTCATAAATCTTCTCAGATCTCTTCCAATTTTCTTTAATTAAAATG |
| At-TCTP1cp | AT3G16640 | CCAAAATTGTAATTTACCGAGAATTGTAAATTTACCTGAAAACCCTACGCTATAGTTTCGACTATAAATACCAAACTTAGGACCTCACTTCAGAATCCCCTCGTCGCTGCGTCTCTCTCCCGCAACCTTCGATTTTCGTTTATTCGCATCCATCGGAGAGAGAAAACAATCAATTAATTAAAATG |
| At-RPL26Acp | AT3G49910 | GAATTAACTTTTACTAGGCCAGAAGTGTAGCTAACATAGAAGAGGCCCATTATAAAACTCTTTAAAATCAAAATCTAAAACAGGCCCAGCCCATTCATAACAAAGCCCTAATATATCGAGTAAACCTAGCTCCACTCAAAACCTAACTATATAACCTTCACACACACTCATAACCTCTTCCTCATCCCCTTAAAAAACCCTAAGAGTAGAGACTCTCTCAATCCCGTTAATTAAAATG |
| At-MED37Ecp | AT5G02500 | ACCAATTTTTGACCGTCCGATGGAAACTCTAGCCTCAACCCAAAACTCTATATAAAGAAATCTTTTCCTTCGTTATTGCTTACCAAATACAAACCCTAGCCGCCTTATTCGTCTTCTTCGTTCTCTAGTTTTTTCCTCAGTCTCTGTTCTTAGATCCCTTGTAGTTTCCAAATCTTTTAATTAAAATG |
| At-AT1G15270 | AT1G15270 | ATACACTTTCAGAGCCCATTTAATAGGTTGCGTTGTTACTACGAACTCATTATAAATATGAACCGTAGCCCCAATCAGAGAGATTCGATACCGTCTGCAACTCTCAGCTACTTTTTCCCCAATTTTGAGCTCAACATCGAACCCTAGCTCAACTTAATTAAAATG |
| At-FKBP12cp | AT5G64350 | TCTGCTTCTTAATTCGGTCTGGTACAGTATTATATTATCCACCTTTGAGAAAGAATAAATAATGGGCCTAAATTTCATCGAATTGGGTTTTGGATTATTGTTAGGCCCAGATAGGGTTTAGATCAAACAGCATGATAATTGATAAATAACAAAATATATAGGCAAAAGCTACTCCGAGATTCGAAGCTGCAAAGAACGCGAAACAGTGAGAGAGACAGAGAGAATTAATTAAAATG |
| At-AT4G25140 | AT4G25140 | CAATTGCATGATGTCTCCATTGACACGTGACTTCTCGTCTCCTTTCTTAATATATCTAACAAACACTCCTACCTCTTCCAAAATATATACACATCTTTTTGATCAATCTCTCATTCAAAATCTCATTCTCTCTAGTAAACAAGTTAATTAAAATG |
| At-DRT112cp | AT1G20340 | ATGCCATGTCACGACACAGTATCTAAAATCAACCAATCACAACGCGTCTTTATAGATAACTTGTTTTTTTATGGAGTTTGCTTTTAGAGCCATCCATTGTCCTATCTCACTTTCTCTCTTTCACCACATAAAAACTCATAAACTCGATCGAACCAAAGCTAAACGAAAAACTTAAAACCCAAATCTTATCACTACTCTAAAAGATTAATTAAAATG |
| At-AT1G13930 | AT1G13930 | TTTTCACATTTACGTCTACAATCACAATGTATGTTATTTAGAACAATAATTATAGTGGCTTAAAAATCATTAATGAAAGTAGATAATAGTATACTTTTTCTTTTTCTTTGTGTGGCCAACATATCCATTTTCTAGTCTATATATACACATATCCATCTCTTAACTCTTCCATCCAAAAAAAACAAAACAAAAAATTATATTCAAGAGAAATTAATTAAAATG |
| At-RPL14Bcp | AT4G27090 | TATTTAGTAAAGATAGGCCCAAACCACAAAACCCTAGAATGAAGATTATATATAGTGCAAAACCTAATCGATTTTTTCCTCTGCTGTCGCTCGTCTACATTTACACTCGGAGCTTAGACCTTCCAATCTACCGTTAATTAAAATG |
| At-PDF2cp | AT2G02130 | TTAAAAATGCAATTCTCTAATAGACTATCAAATATCCCGATACCTCTTTATATAGTGCCATCTTCATCCTTAGTAATGTACACACACACACATAACACTTATTTCCAACTCTGTCTCTCTCAATTTTCTTTCTCTTTTAATTAAAATG |

## Table S2

DNA sequences used to construct the expression cassettes. The coding regions of the mCherry, sTF and example protein genes were codon-optimized for *Nicotiana benthamiana*. In the sequences representing the sTFs, the DNA-binding regions are shown in italics, the shaded sequences represent the nuclear localization signal, and the underlined parts correspond to transcriptional activation domains (VP64 or VP16). In the construct containing eight sTF-specific binding sites (8BS), the binding sites (either Bm3R1 or TetR) are underlined. In the sequences encoding the protein products, the signal sequence (SS) of the Pr1B protein (*) is shown in italics, and the STREP-II tag sequence is underlined in bold.

| **Gene** | **Sequence (5′→3′)** |
| --- | --- |
| Bm3R1-VP64 (sTF) | *ATGGAGAGTACACCAACCAAACAGAAAGCTATTTTTAGCGCAAGCCTGTTATTATTTGCTGAGCGTGGCTTTGACGCTACGACGATGCCCATGATAGCCGAAAATGCTAAAGTTGGGGCTGGAACCATATACCGATACTTCAAAAACAAAGAAAGTCTGGTGAATGAACTGTTTCAACAACACGTAAACGAGTTCTTGCAGTGCATCGAGTCTGGACTCGCTAACGAGCGTGACGGCTATAGAGATGGATTTCATCATATATTTGAGGGCATGGTCACCTTCACAAAAAACCACCCAAGGGCTCTGGGTTTCATAAAAACGCACAGTCAAGGCACATTCCTTACGGAGGAGAGCAGATTAGCATATCAGAAATTAGTGGAGTTCGTATGTACTTTCTTTAGAGAAGGACAGAAGCAAGGAGTTATTCGAAACCTGCCGGAAAACGCCTTAATTGCCATCCTGTTTGGGTCTTTCATGGAAGTTTACGAAATGATAGAGAATGATTACCTCTCCCTTACCGACGAATTGTTGACTGGCGTCGAAGAATCATTGTGGGCAGCATTGTCTAGGCAATCA*GAATTCCCACCTAAGAAGAAAAGGAAAGTATCCGCCTCCGGTTCTGGGCGTGCCGACGCTCTGGACGATTTCGACCTCGATATGTTGGGGTCAGACGCATTGGACGACTTTGATTTGGACATGTTAGGTAGTGATGCTTTAGACGATTTCGACTTGGACATGTTAGGCTCCGATGCATTGGACGATTTTGACTTAGATATGTTGATTAATAGTAGGTAA |
| Bm3R1-VP16 (sTF) | *ATGGAGAGTACACCAACCAAACAGAAAGCTATTTTTAGCGCAAGCCTGTTATTATTTGCTGAGCGTGGCTTTGACGCTACGACGATGCCCATGATAGCCGAAAATGCTAAAGTTGGGGCTGGAACCATATACCGATACTTCAAAAACAAAGAAAGTCTGGTGAATGAACTGTTTCAACAACACGTAAACGAGTTCTTGCAGTGCATCGAGTCTGGACTCGCTAACGAGCGTGACGGCTATAGAGATGGATTTCATCATATATTTGAGGGCATGGTCACCTTCACAAAAAACCACCCAAGGGCTCTGGGTTTCATAAAAACGCACAGTCAAGGCACATTCCTTACGGAGGAGAGCAGATTAGCATATCAGAAATTAGTGGAGTTCGTATGTACTTTCTTTAGAGAAGGACAGAAGCAAGGAGTTATTCGAAACCTGCCGGAAAACGCCTTAATTGCCATCCTGTTTGGGTCTTTCATGGAAGTTTACGAAATGATAGAGAATGATTACCTCTCCCTTACCGACGAATTGTTGACTGGCGTCGAAGAATCATTGTGGGCAGCATTGTCTAGGCAATCA*GAATTCCCACCTAAAAAAAAGAGAAAAGTTTCCACTGCCCCCCCAACGGACGTCTCTCTGGGCGACGAGCTGCACCTCGATGGTGAGGACGTGGCTATGGCTCATGCTGATGCCTTAGACGACTTCGACTTAGATATGCTGGGAGATGGCGACTCACCGGGACCAGGGTTTACACCTCATGATTCTGCTCCTTACGGAGCTTTAGATATGGCCGATTTTGAATTTGAGCAAATGTTCACTGACGCCCTTGGAATAGACGAATACGGGGGCTAA |
| TetR-VP64 (sTF) | *ATGTCAAGATTAGACAAAAGCAAAGTAATCAATAGTGCATTAGAACTTTTAAACGAGGTCGGAATAGAGGGATTAACTACACGTAAACTCGCCCAGAAGCTCGGAGTTGAACAACCTACGCTGTATTGGCATGTTAAAAATAAACGAGCATTATTGGATGCTCTGGCTATTGAGATGCTCGATAGGCACCATACCCACTTTTGCCCTCTGGAGGGGGAATCTTGGCAAGACTTCTTGCGTAACAACGCCAAGTCATTCAGATGTGCCTTGCTGAGTCACCGTGACGGCGCTAAAGTCCATCTCGGAACCCGACCGACCGAGAAGCAATACGAGACCTTAGAAAACCAATTAGCCTTTCTTTGCCAGCAAGGGTTTTCATTAGAGAATGCTCTCTACGCCCTTTCCGCTGTTGGGCATTTCACCCTGGGTTGCGTCTTGGAGGATCAGGAACATCAAGTAGCAAAGGAGGAACGAGAGACACCTACTACGGATTCTATGCCGCCCCTCCTCAGGCAGGCAATTGAACTGTTCGATCATCAGGGAGCTGAACCTGCTTTTCTGTTTGGCCTGGAATTGATAATATGCGGACTGGAGAAACAGTTAAAGTGCGAGAGCGGTAGC*GAATTCCCACCTAAGAAGAAAAGGAAAGTATCCGCCTCCGGTTCTGGGCGTGCCGACGCTCTGGACGATTTCGACCTCGATATGTTGGGGTCAGACGCATTGGACGACTTTGATTTGGACATGTTAGGTAGTGATGCTTTAGACGATTTCGACTTGGACATGTTAGGCTCCGATGCATTGGACGATTTTGACTTAGATATGTTGATTAATAGTAGGTAA |
| TetR-VP16 (sTF) | *ATGTCAAGATTAGACAAAAGCAAAGTAATCAATAGTGCATTAGAACTTTTAAACGAGGTCGGAATAGAGGGATTAACTACACGTAAACTCGCCCAGAAGCTCGGAGTTGAACAACCTACGCTGTATTGGCATGTTAAAAATAAACGAGCATTATTGGATGCTCTGGCTATTGAGATGCTCGATAGGCACCATACCCACTTTTGCCCTCTGGAGGGGGAATCTTGGCAAGACTTCTTGCGTAACAACGCCAAGTCATTCAGATGTGCCTTGCTGAGTCACCGTGACGGCGCTAAAGTCCATCTCGGAACCCGACCGACCGAGAAGCAATACGAGACCTTAGAAAACCAATTAGCCTTTCTTTGCCAGCAAGGGTTTTCATTAGAGAATGCTCTCTACGCCCTTTCCGCTGTTGGGCATTTCACCCTGGGTTGCGTCTTGGAGGATCAGGAACATCAAGTAGCAAAGGAGGAACGAGAGACACCTACTACGGATTCTATGCCGCCCCTCCTCAGGCAGGCAATTGAACTGTTCGATCATCAGGGAGCTGAACCTGCTTTTCTGTTTGGCCTGGAATTGATAATATGCGGACTGGAGAAACAGTTAAAGTGCGAGAGCGGTAGC*GAATTCCCACCTAAAAAAAAGAGAAAAGTTTCCACTGCCCCCCCAACGGACGTCTCTCTGGGCGACGAGCTGCACCTCGATGGTGAGGACGTGGCTATGGCTCATGCTGATGCCTTAGACGACTTCGACTTAGATATGCTGGGAGATGGCGACTCACCGGGACCAGGGTTTACACCTCATGATTCTGCTCCTTACGGAGCTTTAGATATGGCCGATTTTGAATTTGAGCAAATGTTCACTGACGCCCTTGGAATAGACGAATACGGGGGCTAA |
| mCherry | ATGGTAAGCAAGGGAGAAGAGGATAACATGGCAATCATAAAGGAATTTATGCGTTTCAAGGTCCACATGGAAGGTTCTGTCAATGGGCACGAGTTCGAGATTGAAGGCGAGGGGGAGGGTAGACCGTATGAAGGGACCCAGACTGCCAAATTGAAGGTAACAAAAGGCGGGCCGCTTCCATTCGCTTGGGATATCCTCAGTCCGCAGTTCATGTATGGCTCCAAGGCCTATGTGAAGCATCCTGCAGATATACCCGACTATTTAAAGCTCAGTTTCCCCGAGGGCTTCAAATGGGAAAGAGTTATGAATTTTGAGGACGGAGGTGTTGTAACCGTCACGCAGGATAGCAGCTTACAGGACGGCGAATTTATTTACAAGGTAAAGTTGCGTGGTACGAATTTTCCTTCAGATGGTCCGGTCATGCAGAAGAAGACTATGGGTTGGGAAGCAAGCTCTGAGAGGATGTATCCCGAAGATGGGGCTCTTAAAGGCGAGATAAAGCAGAGGCTGAAACTGAAGGACGGCGGGCACTACGATGCCGAAGTCAAAACCACCTATAAGGCTAAAAAGCCCGTACAGCTTCCCGGTGCTTACAACGTGAACATCAAATTAGACATTACCTCCCACAATGAAGACTATACCATCGTGGAGCAATACGAGAGGGCCGAGGGAAGGCACTCTACAGGAGGAATGGATGAACTCTACAAAGGATCCTAA |
| 8-BS (Bm3R1) | GATTTGCAGGCATTTGCTCGGCTAGTCGGAATGAACATTCATTCCGAGACCTAGGATGTGACGGAATGAAGGTTCATTCCGGACTCTAGATAAGCACGGAATGAACTTTCATTCCGCTGAAGCTTGTCAATCGGAATGAAGGTTCATTCCGGCTAGTCGGAATGAACATTCATTCCGAGACCTAGGATGTGACGGAATGAAGGTTCATTCCGGACTCTAGATAAGCACGGAATGAACTTTCATTCCGCTGAAGCTTGTCAATCGGAATGAAGGTTCATTCCGGCTAGTTCTCCCCGGAAACTG |
| 8-BS (TetR) | GATTTGCAGGCATTTGCTCGGCTAGCTCTCTATCACTGATAGGGAGTATTGACAAGCTTTCTCTATCACTGATAGGAGTGGCTTATCTAGATCTCTATCACTGATAGGGAGTTCACATCCTAGGTCTCTATCACTGATAGGGAGTACTAGCTCTCTATCACTGATAGGGAGTATTGACAAGCTTTCTCTATCACTGATAGGAGTGGCTTATCTAGATCTCTATCACTGATAGGGAGTTCACATCCTAGGTCTCTATCACTGATAGGGAGTACTAGTTCTCCCCGGAAACTG |
| At-term #1 (AT2G06520)** | TATATCTTTCTTACATCATTATTGTAATCTGTTCTCCTTCTGTGTATTCGTTTCAATGTTGCAGCAATGAACTTTTGGATAAAAGTCAAATTTGTTGTTTCCTTAATTCGAAAGACGATTGAGACTTGAAATCATAACACTAAGCTTCATTGAATCAAGATTCAATAGTATTCATCAATTCATAATATAATAGTGTACTAAACTCGAGCTTGCATATTCTGAGTTAATTGAAATACCTCACTGTAATACCTAGAACGAACTTACCTTACGAGCAAATCAAGCATGTATTTACTCTCGGATGTATAATTCACCTTATCAACCTTCACAACAGTCATCTTCACTCTTTGTTCATCCCCATACGATTCCTCTTTGATCTTCAGCTTC |
| At-term #2 (AT3G15353)*** | TCTTTAATCAAAATGTAATATGAATAAAAGTTGATGTGGGCTCATCTATTGAGCTCATGTCTCTCTTATTACTACTCTCTAGTATGGTGTGATGTAATGGGTTATGACCCTTCTTTCCCTTCCCTATAAAACTAAAGAAACTTGCAAGATAATTGAAAAGATGGTTTCTTTTTATTATCAATCGCATCAAAATGGGATTTTGTATCAAATGCATACATTATCTCTTGCTTTTATACCCTAAACCCATACCGGGTGATGAACAATCTTCTGTTGCTCATTCCTTTTGATGATCCATCAAATTACGTATTAGAAAAAGAAAAAAAAGTATCAGACGTTGAAACCTTCTGCTCGGAGACAAATTTTATGAGCCTCGATCC |
| 35S (2x) promoter | AAGCTTGCCAACATGGTGGAGCACGACACTCTCGTCTACTCCAAGAATATCAAAGATACAGTCTCAGAAGACCAAAGGGCTATTGAGACTTTTCAACAAAGGGTAATATCGGGAAACCTCCTCGGATTCCATTGCCCAGCTATCTGTCACTTCATCAAAAGGACAGTAGAAAAGGAAGGTGGCACCTACAAATGCCATCATTGCGATAAAGGAAAGGCTATCGTTCAAGATGCCTCTGCCGACAGTGGTCCCAAAGATGGACCCCCACCCACGAGGAGCATCGTGGAAAAAGAAGACGTTCCAACCACGTCTTCAAAGCAAGTGGATTGATGTGAACATGGTGGAGCACGACACTCTCGTCTACTCCAAGAATATCAAAGATACAGTCTCAGAAGACCAAAGGGCTATTGAGACTTTTCAACAAAGGGTAATATCGGGAAACCTCCTCGGATTCCATTGCCCAGCTATCTGTCACTTCATCAAAAGGACAGTAGAAAAGGAAGGTGGCACCTACAAATGCCATCATTGCGATAAAGGAAAGGCTATCGTTCAAGATGCCTCTGCCGACAGTGGTCCCAAAGATGGACCCCCACCCACGAGGAGCATCGTGGAAAAAGAAGACGTTCCAACCACGTCTTCAAAGCAAGTGGATTGATGTGATATCTCCACTGACGTAAGGGATGACGCACAATCCCACTATCCTTCGCAAGACCCTTCCTCTATATAAGGAAGTTCATTTCATTTGGAGAGGACACGCTGAAATCACCAGTCTCTCTCTACAAATCTATCTCTC |
| SS_GOX_  STREP | *ATGGGATTTTTTCTCTTTTCACAAATGCCCTCATTTTTTCTTGTCTCTACACTTCTCTTA*TTCCTAATAATATCTCACTCTTCTCATGCCTCTAGAGGTACGTCTAACGGAATTGAGGCTTCTCTTTTGACTGATCCAAAGGATGTTTCTGGAAGGACTGTGGATTACATTATTGCTGGTGGAGGACTTACTGGTCTTACTACTGCTGCTAGGCTTACTGAGAACCCAAACATTTCTGTGCTTGTGATTGAGTCTGGATCTTACGAATCTGATAGGGGACCAATTATCGAGGATCTCAACGCTTACGGTGATATTTTCGGATCTTCCGTGGATCATGCTTATGAGACTGTGGAGCTTGCTACTAACAATCAGACTGCTCTTATTAGGTCTGGAAACGGACTTGGAGGATCTACTCTTGTTAACGGTGGAACTTGGACTAGACCACATAAGGCTCAAGTTGATTCTTGGGAGACTGTTTTCGGAAATGAGGGATGGAATTGGGATAATGTGGCTGCTTACTCTCTTCAAGCTGAAAGAGCTAGGGCTCCAAACGCTAAGCAAATTGCTGCTGGACATTACTTCAATGCTTCATGCCATGGTGTTAACGGAACTGTTCATGCTGGACCAAGAGATACTGGTGATGATTACTCCCCAATTGTGAAGGCTCTTATGTCTGCTGTTGAGGATAGAGGTGTGCCAACTAAGAAGGATTTTGGTTGCGGAGATCCACATGGTGTTTCCATGTTCCCAAACACTCTTCATGAGGATCAGGTTAGATCAGATGCTGCTAGAGAATGGCTTCTTCCAAATTACCAGAGGCCAAACCTTCAAGTTCTTACTGGACAGTACGTTGGAAAGGTGTTGCTTTCTCAGAATGGAACTACACCAAGAGCTGTTGGAGTTGAGTTCGGAACTCACAAGGGAAATACTCACAACGTTTACGCTAAGCACGAAGTTCTTTTGGCTGCTGGATCTGCTGTTTCTCCAACTATTCTCGAATACTCCGGAATTGGAATGAAGTCTATCCTTGAGCCACTCGGAATTGATACTGTGGTTGATCTTCCAGTGGGACTTAATCTTCAGGATCAGACTACTGCTACTGTGAGGTCTAGGATTACTTCTGCTGGTGCTGGACAAGGACAAGCTGCTTGGTTTGCTACTTTCAACGAGACTTTCGGAGATTATTCTGAGAAGGCTCACGAGCTTTTGAACACTAAGCTTGAGCAATGGGCTGAAGAAGCTGTTGCTAGGGGAGGATTCCATAACACTACTGCACTCCTCATTCAGTACGAGAATTACAGGGATTGGATCGTTAACCACAATGTGGCTTACTCTGAGCTTTTCCTCGATACTGCTGGTGTTGCTTCTTTCGATGTGTGGGATCTTTTGCCTTTTACTAGGGGTTACGTGCACATTCTTGATAAGGACCCATACCTTCACCATTTCGCTTACGATCCTCAATACTTCCTTAACGAGCTTGATCTTCTTGGTCAAGCTGCTGCTACTCAACTTGCTAGGAACATTTCTAACTCCGGTGCTATGCAAACTTATTTCGCTGGTGAGACTATTCCAGGTGATAACCTCGCTTACGATGCTGATCTTTCTGCTTGGACTGAGTACATTCCATACCACTTCAGGCCAAATTATCACGGTGTTGGAACTTGTTCTATGATGCCAAAAGAAATGGGAGGTGTGGTTGATAATGCTGCTAGGGTTTACGGTGTTCAAGGACTTAGAGTGATCGATGGATCGATTCCACCAACTCAGATGTCATCTCACGTGATGACTGTGTTTTACGCTATGGCTCTCAAGATTTCCGATGCTATCCTTGAGGATTACGCTTCTATGCAG**TGGTCACATCCTCAATTTGAGAAG**AAAGATGAACTTTGA |
| protA_  STREP | ATGGGAAATGCTGCACAGCATGATGAAGCTCAACAGAACGCATTCTACCAAGTTCTTAACATGCCTAATTTGAACGCTGATCAGAGAAATGGTTTCATTCAATCTCTTAAGGATGATCCATCTCAGTCAGCTAACGTGTTAGGAGAAGCACAAAAGCTCCAGGATTCACAAGCTCCAAAAGCTGATGCACAACAGAATAAGTTTAACAAAGATCAACAGAGTGCATTCTACGAGATCCTCAACATGCCTAATCTCAACGAAGAGCAGAGAAACGGTTTTATCCAATCTCTTAAAGATGATCCAAGTCAGTCTACTAATGTTCTTGGAGAAGCTAAGAAATTGCAGGAGTCACAAGCTCCTAAGGCAGATAATAACTTCAATAAGGAACAGCAAAACGCATTCTACGAGATCTTGAATATGCCTAATTTGAACGAAGAGCAGAGGAACGGTTTCATCCAAAGTCTTAAAGATGATCCTTCACAAAGTGCTAATCTTTTGGCTGAAGCAAAGAAATTGCAGGAGTCTCAAGCTCCAAAGGCAGATAATAAGTTTAACAAAGAACAACAGAACGCTTTCTACGAGATTCTTCATTTGCCTAATCTTAACGAAGAGCAGAGAAATGGTTTTATTCAATCACTTAAAGATGATCCTTCTCAGTCAGCTAATCTTCTCGCTGAGGCAAAGAAATTGAACGATGCTCAAGCACCTAAGGCTGACAATAAGTTTAACAAAGAGCAACAGAATGCATTCTACGAGATTTTACACCTCCCTCAGTTGACAGAAGAGCAAAGGAATGGTTTTATTCAAAGTCTTAAGGATGATCCAAGTGTGTCTAAAGAAATCTTAGCAGAAGCAAAGAAATTGAATGATGCT**TGGTCCCACCCTCAGTTCGAGAAG**AAGGATGAGCTTTGA |
| SS_VEGF165_STREP | *ATGGGATTTTTTCTCTTTTCACAAATGCCCTCATTTTTTCTTGTCTCTACACTTCTCTTA*TTCCTAATAATATCTCACTCTTCTCATGCCTCTAGAGGTACGGGAGCACCAATGGCTGAAGGAGGAGGTCAAAATCACCACGAAGTTGTTAAGTTTATGGATGTTTACCAAAGGAGTTACTGTCACCCTATTGAAACACTTGTTGATATCTTCCAAGAGTACCCTGATGAAATTGAGTACATCTTCAAGCCTTCATGCGTTCCACTTATGAGATGTGGTGGATGTTGCAATGATGAAGGTTTGGAGTGCGTGCCTACTGAAGAGAGTAACATCACAATGCAGATTATGAGAATCAAGCCACATCAGGGTCAGCACATCGGAGAAATGTCTTTTCTTCAACATAATAAGTGTGAGTGCAGGCCTAAGAAAGATAGAGCTAGGCAGGAAAACCCTTGCGGACCATGTTCTGAGAGAAGGAAACACCTTTTCGTGCAAGATCCACAGACCTGCAAGTGTTCTTGTAAGAATACTGATTCAAGGTGTAAGGCAAGGCAGTTAGAGTTGAACGAGAGGACCTGTAGATGCGATAAGCCAAGAAGA**TGGTCCCACCCTCAGTTCGAGAAG**AAGGATGAGCTTTGA |
| SS_mCherry | *ATGGGATTTTTTCTCTTTTCACAAATGCCCTCATTTTTTCTTGTCTCTACACTTCTCTTA*TTCCTAATAATATCTCACTCTTCTCATGCCTCTAGAGGTACGGTAAGCAAGGGAGAAGAGGATAACATGGCAATCATAAAGGAATTTATGCGTTTCAAGGTCCACATGGAAGGTTCTGTCAATGGGCACGAGTTCGAGATTGAAGGCGAGGGGGAGGGTAGACCGTATGAAGGGACCCAGACTGCCAAATTGAAGGTAACAAAAGGCGGGCCGCTTCCATTCGCTTGGGATATCCTCAGTCCGCAGTTCATGTATGGCTCCAAGGCCTATGTGAAGCATCCTGCAGATATACCCGACTATTTAAAGCTCAGTTTCCCCGAGGGCTTCAAATGGGAAAGAGTTATGAATTTTGAGGACGGAGGTGTTGTAACCGTCACGCAGGATAGCAGCTTACAGGACGGCGAATTTATTTACAAGGTAAAGTTGCGTGGTACGAATTTTCCTTCAGATGGTCCGGTCATGCAGAAGAAGACTATGGGTTGGGAAGCAAGCTCTGAGAGGATGTATCCCGAAGATGGGGCTCTTAAAGGCGAGATAAAGCAGAGGCTGAAACTGAAGGACGGCGGGCACTACGATGCCGAAGTCAAAACCACCTATAAGGCTAAAAAGCCCGTACAGCTTCCCGGTGCTTACAACGTGAACATCAAATTAGACATTACCTCCCACAATGAAGACTATACCATCGTGGAGCAATACGAGAGGGCCGAGGGAAGGCACTCTACAGGAGGAATGGATGAACTCTACAAAGATGAGCTTTGA |

*Amino acids 1–30 of *Nicotiana tabacum* pathogenesis-related protein 1B (accession no: XP_016487756.1)

**The 3′-UTR + terminator region of the *Arabidopsis thaliana* gene (transcript ID) AT2G06520.1 (https://phytozome-next.jgi.doe.gov/info/Athaliana_Araport11)

***The 3′-UTR + terminator region of the *A. thaliana* gene (transcript ID) AT3G15353.1 (https://phytozome-next.jgi.doe.gov/info/Athaliana_Araport11)

## Table S3

Primers used to construct the expression cassettes

| **Primer number** | **Target** | **Use of the PCR product** | **Primer name** | **Sequence (5′→3′)** |
| --- | --- | --- | --- | --- |
| 1925 | 8BS(Bm3R1) | Intermediate construct for Bm3R1-based SES | 8BS_Nb_GA_F | GAAACAGCTATGACCATGATTACGCCAGGCGCGCCGAATTAACCCTC |
| 1926 |  |  | 8BS_Nb_BMR_GA_R | CTTTCTGTTTGGTTGGTGTACTCTCCATTTTAATTAACAGTTTCCGGGGAGAACTAG |
| 1925 | 8BS(TetR) | Intermediate construct for TetR-based SES | 8BS_Nb_GA_F | GAAACAGCTATGACCATGATTACGCCAGGCGCGCCGAATTAACCCTC |
| 1927 |  |  | 8BS_Nb_Tet_GA_R | CTTTGCTTTTGTCTAATCTTGACATTTTAATTAACAGTTTCCGGGGAGAACTAG |
| 1973 | At_TCTP1 core promoter | CP for Bm3R1 in SES_B64-A; SES_B16-A; SES_B64-B | At_TCTP1_GA1_F | TGATCTTCAGCTTCATTTAAATGCGCCAAAATTGTAATTTACCGAGAATTGTAAATTTAC |
| 1974 |  |  | At_TCTP1_GA1_R | GTTTGGTTGGTGTACTCTCCATTTTAATTAATTGATTGTTTTCTCTCTCCGATGGATG |
| 1973 | At_TCTP1 core promoter | CP for TetR in SES_T64-A; SES_T16-A; SES_T16-B; | At_TCTP1_GA1_F | TGATCTTCAGCTTCATTTAAATGCGCCAAAATTGTAATTTACCGAGAATTGTAAATTTAC |
| 1975 |  |  | At_TCTP1_GA2_R | TGCTTTTGTCTAATCTTGACATTTTAATTAATTGATTGTTTTCTCTCTCCGATGGATG |
| 1976 | AT1G15270 core promoter | CP for mCherry in SES_B64-B; SES_T16-B; | AT1G15270_GA_F | CTAGTTCTCCCCGGAAACTGATACACTTTCAGAGCCCATTTAATAGG |
| 1977 |  |  | AT1G15270_GA_R | CCTCTTCTCCCTTGCTTACCATTTTAATTAAGTTGAGCTAGGGTTCG |
| 1978 | At_MT2B core promoter | CP for mCherry in SES_B64-A; SES_B16-A; SES_T64-A; SES_T16-A; | At_MT2B_GA_F | CTAGTTCTCCCCGGAAACTGGTGGACAAAGATCGTTGACACG |
| 1979 |  |  | At_MT2B_GA_R | CCTCTTCTCCCTTGCTTACCATTTTAATTAAAGAAAATTGGAAGAGATCTGAGAAGAT |
| 1930 | At_RPL41D core promoter | CP for Bm3R1 in SES_B64-C | At_002cp_GA_F | TGATCTTCAGCTTCATTTAAATGCGATCCCCTCTGGCAAATTCTTATCC |
| 1931 |  |  | At_002cp_GA_R | TCTGTTTGGTTGGTGTACTCTCCATTTTAATTAAGCAGAAGCTGTCTCC |
| 1928 | At_ATTI7 core promoter | CP for mCherry in SES_B64-C | At_003cp_GA_F | CTAGTTCTCCCCGGAAACTGGAATTTGTGGTTCTCGTGAAGTCG |
| 1929 |  |  | At_003cp_GA_F | ATCCTCTTCTCCCTTGCTTACCATTTTAATTAACTTTGTCTCTGTCTTTTTCTCTGTCTC |
| 1925 | 1-4×BS (Bm3R1) region | 1-4×BS for SES_B64-A (1×; 2×; 4× BS) | 8BS_Nb_GA_F | GAAACAGCTATGACCATGATTACGCCAGGCGCGCCGAATTAACCCTC |
| 2064 |  |  | 1-4BS_Nb_GA_R | TGTCCACCAGTTTCCGGGGAGAACTAGC |
| 1925 | 0-BS region | 0×BS for SES_B64-A (0× BS) | 8BS_Nb_GA_F | GAAACAGCTATGACCATGATTACGCCAGGCGCGCCGAATTAACCCTC |
| 2065 |  |  | 0BS_Nb_GA_R | TGTCCACCAGTTTCCGGGGAGAACCTAG |
| 2066 | At_MT2Bcp-mCherry | Fragment for SES_B64-A (0×; 1×; 2×; 4× BS) | Nb_cp5_mCh_GA_F | GTTCTCCCCGGAAACTGGTGGACAAAG |
| 2067 |  |  | Nb_cp5_mCh_GA_R | GTAAGAAAGATATATAGCTATTAGGATCCTTTGTAGAG |
| 2000 | mCherry + AT2G06520 terminator | Fragment for 35S(2×) pr. control | Nb_mCh_GA_F | GTCTCTCTCTACAAATCTATCTCTCTTAATTAAAATGGTAAGCAAGGGAGAAG |
| 2001 |  |  | Nb_mCh_GA_R | GGCGATGGCCCACTACGTACCCGGGCGCATTTAAATGAAGCTGAAGATC |
| 2352 | 4× or 8× BS (Bm3R1) region | Fragments for SES_B64-A (12×; 16× BS) | Nb_xBS_GA_F | ACTGGTGGACAAAGATCGTTGACACATTTGCAGGCATTTGCTCGGC |
| 2353 |  |  | Nb_mCh_GA_R | GATGTAAGAAAGATATATAGCTATTAGGATCC |
| 2115 | GOX-CDS | For cytosolic GOX | Nb_GOX_GA_F | CAGATCTCTTCCAATTTTCTTTAATTAAAATGTCTAACGGAATTGAGGCTTCTCTTTTG |
| 2117 |  |  | Nb_GOX_GA_R | ATATATAGCTATTAGGATCCTCACTTCTCAAATTGAGGATGTGACCACTGCATAGAAGCGTAATCCTCAAGG |
| 2118 | VEGF165-CDS | For cytosolic VEGF | Nb_VEGF165_GA_F | CAGATCTCTTCCAATTTTCTTTAATTAAAATGGCACCAATGGCTGAAGGAGGA |
| 2119 |  |  | Nb_VEGF165_GA_R | GAAAGATATATAGCTATTAGGATCCTCACTTCTCAAATTGAGGATGTGACCATCTTCTTGGCTTATCGCATCTACAG |
| 2120 | AT2G06520 terminator + At_TCTP1 core promoter | For cytosolic GOX and VEGF | Nb_ter_CP_GA_F | GGATCCTAATAGCTATATATCTTTCTTACATC |
| 2121 |  |  | Nb_ter_CP_GA_R | GCTTTCTGTTTGGTTGGTGTACTCTC |
| 2150 | KDEL-AT2G06520 terminator + At_TCTP1cp | Fragment for ER-targeted GOX | KDEL_Nb_ter_GA_F | CAATTTGAGAAGAAAGATGAACTTTGAGGATCCTAATAGCTATATATCTTTCTTAC |
| 2151 |  |  | KDEL_Nb_ter_GA_R | GGTTGGTGTACTCTCCATTTTAATAAATTGATTGTTTTCTCTCTCCGATGGATGCG |
| 2152 | 3’-part of SS-GOX-CDS | Fragment for ER-targeted GOX | GOX_KDEL_GA_F | GAGTTCGGAACTCACAAGGGAAATACTC |
| 2153 |  |  | GOX_KDEL_GA_R | CAAAGTTCATCTTTCTTCTCAAATTGAGGATGTGACCACTGC |
| 2154 | 5’-part of SS-GOX-CDS | Fragment for ER-targeted GOX | SS_GOX_GA_F | CTCAGATCTCTTCCAATTTTCTTTAATTAAAATGGGATTTTTTCTCTTTTCACAAATGCC |
| 2155 |  |  | SS_GOX_GA_R | GAGTATTTCCCTTGTGAGTTCCGAACTC |
| 2156 | 8× BS (Bm3R1) + At_MT2Bcp + Pr1B-SS | Fragment for ER-targeted VEGF and protA | 8BS_SS_GA_F | GAAACAGCTATGACCATGATTACGCC |
| 2157 |  |  | 8BS_SS_GA_R | CGTACCTCTAGAGGCATGAGAAGAGTG |
| 2158 | VEGF165 CDS | Fragment for ER-targeted VEGF | SS_VEGF_GA_F | CTCTTCTCATGCCTCTAGAGGTACGGGAGCACCAATGGCTGAAGGAG |
| 2159 |  |  | SS_VEGF_GA_R | GAAAGATATATAGCTATTAGGATCCTCAAAGCTCATCCTTCTTCTCGAAC |
| 2160 | Protein A CDS | Fragment for ER-targeted protA | SS_protA_GA_F | CTCTTCTCATGCCTCTAGAGGTACGGGAAATGCTGCACAGCATGATGAAG |
| 2161 |  |  | SS_protA_GA_R | GAAAGATATATAGCTATTAGGATCCTCAAAGCTCATCCTTCTTCTCGAAC |
| 2161 | Protein A CDS | Fragment for cytosolic protA | SS_protA_GA_R | GAAAGATATATAGCTATTAGGATCCTCAAAGCTCATCCTTCTTCTCGAAC |
| 2164 |  |  | protA_GA_F | CTCAGATCTCTTCCAATTTTCTTTAATTAAAATGGGAAATGCTGCACAGCATGATG |
| 2491 | 8× BS (Bm3R1) region | Fragment for SES_B64-A transfer into pK2GW7.0 | Nb_K2GW_SES_GA1_F | CCTTAATTCTCATGTATGATAATTCGAGCTGGCGCGCCGAATTAACCCTCACTAAAGG |
| 2492 |  |  | Nb_K2GW_SES_GA1_R | GCATTTAAATGAAGCTGAAGATCAAAGAGGAATCG |
| 2493 | Nb_BMR-VP64 region | Fragment for SES_B64-A transfer into pK2GW7.0 | Nb_K2GW_SES_GA2_F | CGATTCCTCTTTGATCTTCAGCTTCATTTAAATGC |
| 2494 |  |  | Nb_K2GW_SES_GA2_R | CTTGGGCCCGACGTCGCATGCCTGCAGCTTGATTTAAATGGATCGAGGCTC |

## Table S4

Primers and gene targets used for transcript analysis.

| Primer number | Target  (amplicon length) | ID in database | Primer name | Sequence (5′→3′) |
| --- | --- | --- | --- | --- |
| 1936 | mCherry  (180 bp) | Synthetic gene  codon optimized for *N. benthamiana* | Nb_mCherry_qPCR_F | GAAGAAGACTATGGGTTGGGA |
| 1937 |  |  | Nb_mCherry_qPCR_R | GATGTTCACGTTGTAAGCACC |
| 1932 | Bm3R1-sTF  (206 bp) | Synthetic gene codon optimized for *N. benthamiana* | Nb_BMR_qPCR_F | CACAGTCAAGGCACATTCC |
| 1933 |  |  | Nb_BMR_qPCR_R | CAATTCGTCGGTAAGGGAGAG |
| 2052 | ribulose bisphosphate carboxylase  (181 bp) | Transcript ID in NbGT*: Nbv6.1trP55774 | Nb_RBC-SC_qPCR_F | GATACTATGACGGCAGATACTGGA |
| 2053 |  |  | Nb_RBC-SC_qPCR_R | GGCTTGTAGGCAATGAAACTG |
| 2080 | UBC-e2 10  (219 bp) | Transcript ID in NbGT*: Nbv6.1trP30205 | Nb_UBC-e2_qPCR_F | AATATGCCATGGGTTAGTTGCTG |
| 2081 |  |  | Nb_UBC-e2_qPCR_R | GCTTCACAAATATGAGAGGCCA |
| 890 | mCherry  (211 bp) | Synthetic gene used in yeast SES_A | mCherry_qPCR_F | GTGATGAACTTCGAGGACGG |
| 891 |  |  | mCherry_qPCR_R | TTCAGCCTCTGCTTGATCTC |
| 484 | *IPP1*  (153 bp) | *S. cerevisiae* native / reference gene | Sc_IPP1_qPCR_F | ACTTTGAACCCAATCATCCA |
| 485 |  |  | Sc_IPP1_qPCR_R | CACCAACTGCCTTAGTTTCTG |

*NbGT = Nicotiana benthamiana Genome & Transcriptome Database (<http://benthgenome.qut.edu.au/>)
